# Intratumoral tertiary lymphoid structures promote patient survival and immunotherapy response in head neck squamous cell carcinoma

**DOI:** 10.1007/s00262-022-03310-5

**Published:** 2022-12-08

**Authors:** Zhonglong Liu, Xiaoyan Meng, Xiao Tang, Weili Zou, Yue He

**Affiliations:** 1 Department of Oral Maxillofacial & Head and Neck Oncology, Shanghai Ninth People’s Hospital Affiliated to Shanghai Jiao Tong University School of Medicine, National Center of Stomatology, National Clinical Research Center for Oral Disease, Shanghai, 200011 China; 2 Suzhou Lingdian Biotechnology Co., Ltd, Suzhou, China

**Keywords:** Tertiary lymphoid structures (TLSs), HNSCC, Immune landscape, TME classification, Prognosis, Immunotherapy

## Abstract

Tertiary lymphoid structures (TLSs) hold the potential role in the prediction of immunotherapy response in several clinical trials. TLSs in head neck squamous cell carcinoma (HNSCC) have been investigated through IHC analysis, whereas there is no TLS gene signature to evaluate the level of TLS neogenesis. We here proposed a TLS signature containing 13 chemokines and determined TLS-hi and TLS-low groups in HNSCC samples from The Cancer Genome Atlas. TLS-hi condition signified a better overall survival. A more inflamed immune infiltrative landscape was identified in the TLS-hi tumors characterized by higher proportion of T cells, TCR/BCR activation and antigen processing. High level of TLSs has a determined role in the clinical significance of T cells. Interesting discovery was that innate lymphoid cells and cancer-associated fibroblasts were positively associated with TLS neogenesis in TME of HNSCC. Furthermore, by integrated TLSs with stromal cells and score, immune cells and score, TMB and malignant cells, we proposed a novel HNSCC TME classifications (HNSCC-TCs 1–5), unravelling the counteracted role of stromal cells and score in inflamed immune landscape, which may provide a novel stromal targeted modality in HNSCC therapy. Finally, we verified that TLS statue is an ideal predictor for immune checkpoint blockade immunotherapy. Current study indicated that the TLSs serve as a novel prognostic biomarker and predictor for immunotherapy, which may provide directions to the current investigations on immunotherapeutic strategies for HNSCC.

**Supplementary Information:**

The online version contains supplementary material available at 10.1007/s00262-022-03310-5.

## Introduction

Head and neck squamous cell carcinoma (HNSCC), with 1,000,000 new cases annually, is the sixth most common cancer globally [1]. The comprehensive and sequential therapies for HNSCC include surgery, chemotherapy, radiotherapy and biotherapy. Despite the implementation of this combined therapeutic strategy, a high proportion (50–60%) of advanced HNSCC patients experience relapse or lymph node/distant metastasis [2, 3]. The emergence and development of immunotherapy are represented by the Keynote-048 and 012 clinical trials, proposing the adoption of pembrolizumab (PD-1 antibody) plus chemotherapy as the first-line regimen for recurrent or metastatic HNSCC [4, 5]. These randomized phase 3 clinical trials launched an era of novel immune checkpoint blockade (ICB) therapy for HNSCC. Investigators have paid substantial efforts to identify the influencing factors and predictors for immunotherapeutic efficacy, thus augmenting the survival rate of cancer patients. Through ICB-treated melanoma cohort exploration, the potential role of tertiary lymphoid structures (TLSs) in response to immune therapy has been determined. The co-occurrence of TLSs neogenesis and CD8^+^ T cells/CD20^+^ B cells showed the best clinical survival outcomes among metastatic melanoma patients [6, 7]. In 608 soft tissue sarcoma samples, TLSs were conjugated with the tumor microenvironment (TME) parameters to propose a novel sarcoma immune classes (SICs): A, immune desert; C, vascularized; E, immune and TLS high; B and D, immune-low and high profiles, respectively [8]. This discovery indicates that analysis of TLSs may be applied to guide clinical decision-making and promote the development of precise therapeutic regimens. Although immunohistochemistry (IHC) is the standard approach for detecting TLSs, there is an increasing demand for a robust method to evaluate TLS level based on RNA-sequencing or transcriptomic data from tumor biopsies or resected samples. A gene signature comprising *CCL2*, *CCL4*, *CCL5*, *CCL8, CCL18, CCL19, CCL21, CXCL9, CXCL10, CXCL11*, and *CXCL13* was established and further used to categorize tumors as TLS + or TLS − , and TLSs + tumors were associated with a lower risk for early hepatocellular carcinoma recurrence [9]. Pan-cancer analysis revealed an extremely heterogeneous TLS signature distribution among different cancer types [10]. This prompted us to establish an appropriate TLS signature for HNSCC, thus evaluating TLS neogenesis at the transcript level and contributing to subsequent clinical studies. Recently, TLSs in HNSCC have been investigated through IHC analysis and verified to be associated with decreased *p53* and *Ki-67* scores, enriched filtration of CD8 + cytotoxic T cells and CD57 + NK cells, and improved survival rates[11, 12] However, no in-depth study has been conducted with regard to molecular mechanism and functional diversity between different TLS subtypes.

The aim of the current investigation was primarily to identify a TLS gene signature and evaluate its enrichment status and prognostic prediction role based on transcriptomic data and to explore the diversity of the immune infiltrative landscape and the mechanism through which TLSs contribute to the immune microenvironment. Moreover, we proposed a novel classification approach for HNSCC based on immune and stromal scores, TILs and TLSs, which is more appropriate than the current classifications. Finally, we integrated TLS data with mutation and copy number data to illuminate the driver genes that may be influenced by TLS status. These discoveries may facilitate ongoing clinical trials focusing on the outcome prediction and evaluation.

## Results

### Establishment and validation of TLSs signature for HNSCC

**Fig. 1:**
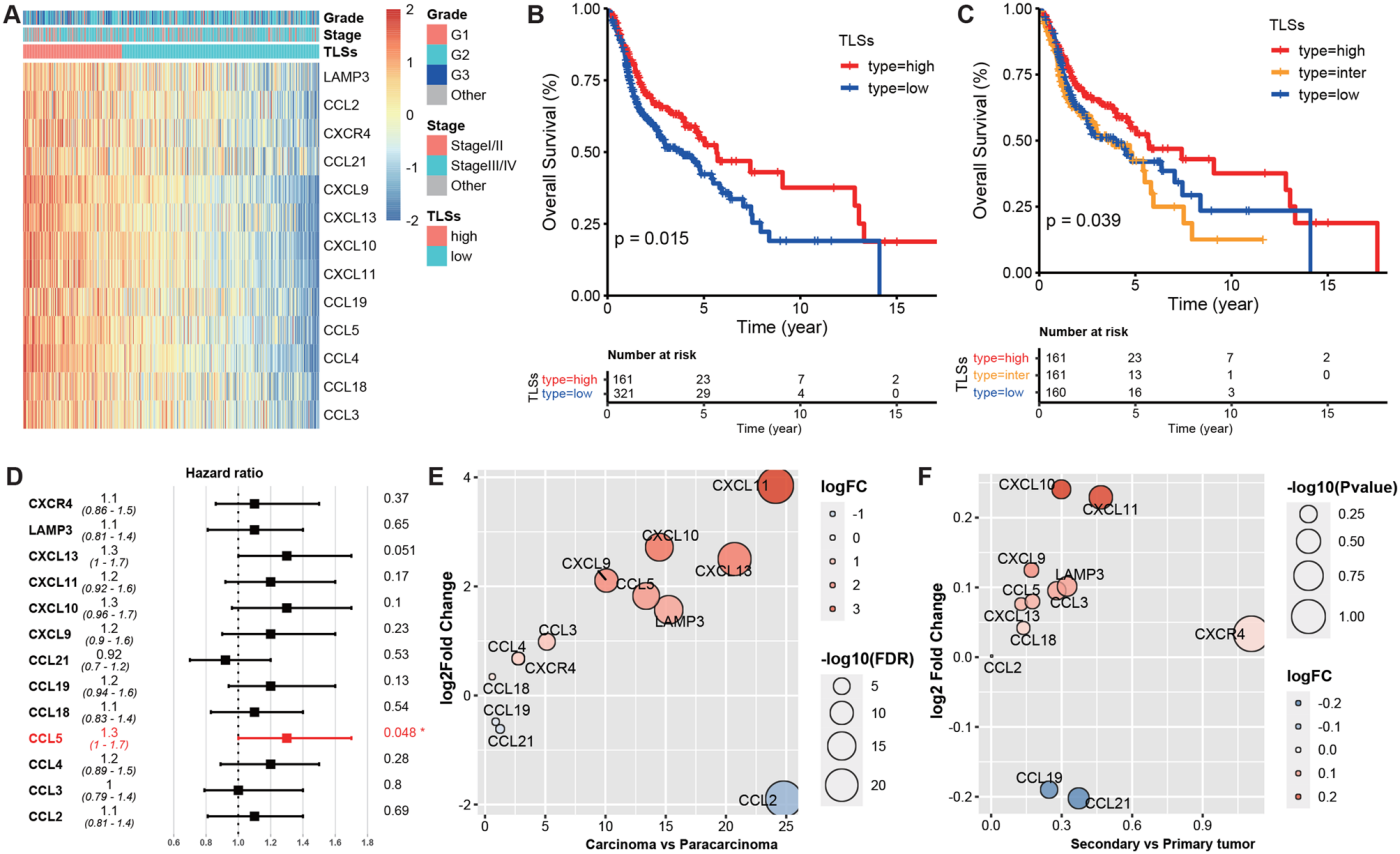
Establishment of TLSs signature in HNSCC samples. **A** Expression profile of 13 chemokines according to the stratification of TLSs statue, grade and stage. **B**, **C** Overall survival analysis among TLS-hi, TLS-inter and TLS-low groups. **D** Univariate analysis of 13 chemokines with regard to the contribution to prognosis. **E** Gene expression of 13 chemokines between carcinoma and paracarcinoma tissues. **F** Gene expression of 13 chemokines between secondary and primary tumors (dataset: TCGA-HNSCC, GSE68858)

Derived from 12 chemokine gene signatures [13, 14] and 9 marker genes [6], we proposed a novel gene signature composed of 13 chemokine genes (*LAMP3, CCL2, CCL3, CCL4, CCL5, CCL18, CCL19, CCL21, CXCL9, CXCL10, CXCL11, CXCK13, CXCR4*) to specifically evaluate HNSCC TLS at the transcriptomic level from the dataset of TCGA (HNSCC) (Fig. 1A). We excluded *BCL6* as its expression was not in accordance with that of the other genes in HNSCC samples (Supplementary Fig. 1A, B). Subsequently, the TLS score was calculated and divided into high and low for each HNSCC tumor specimen in TCGA. As shown in Fig. 1A, this stratification method was powerful enough to distinguish the signature gene expression and provide an easier way to determine the correlation between clinical stage and pathological grade among TLS subtypes. We then probed into the predicative role of TLS signature in HNSCC prognosis with the finding that TLS-hi tumors were associated with better overall survival (OS) rates (*p* < 0.05) than TLS-inter and TLS-low tumors (Fig. 1 B, C). However, there was no prognostic significance in disease-free survival (DFS), recurrence-free survival (RFS) or metastasis-free survival (MFS) rates among different TLS subtypes (Supplementary Fig. 1C–E). The positive correlation of TLS status with HNSCC prognosis was further verified in other two datasets and the merge data of TCGA-HNSCC with CPTAC-HNSCC (Supplementary Fig. 1F–H). To further confirm the predictive role of TLS status in overall survival for HNSCC, we integrated TLSs with other relevant risk factors, such as stage, oropharynx, HPV status, gender and age. In univariate analysis, TLSs (high), stage (I/II) and gender (female) were found to be positive associated with favorable survival for HNSCC. These discoveries were further confirmed by multivariate Cox regression model. HPV status failed to prove its predictive role in better OS for HNSCC in both univariate and multivariate analysis (Supplementary Fig. 2A, B).

Subsequently, we investigated the contribution ratio of every chemokine in TLSs to the prediction of OS rates. Interestingly, no single chemokine showed a significant correlation with favorable OS except for *CCL5* (*p* = 0.048) (Fig. 1D), indicating a synergistic effect of the TLS signature instead of individual chemokines on prognostic prediction. To explore the expression of 13 chemokines in TME, HNSCC single-cell RNA-Seq data [15] were used to identify the major cell types contributing to the TLSs signature, which was composed of CD8/CD4 T cells, NK, B cells, monocyte, macrophage, dendritic, fibroblasts and endothelial (Supplementary Fig. 3A, B). Due to the heterogeneity of TLSs among different cancer types, we tried to identify whether this heterogeneity also exists between normal and tumor tissues, recurrent and primary tumors. Compared to paracarcinoma tissues, carcinoma tissues had higher expression levels of *CXCL9, CXCL10, CXCL11, CXCL13, CCL3, CCL4, CCL5* and *LAMP3*, resulting in higher TLS scores (*p* < 0.001) (Fig. 1E, Supplementary Fig. 2D). A similar phenomenon was observed in the secondary versus primary groups, characterizing by higher level of chemokines except for CCL2, CCL19 and CCL21, even though with no statistical difference in TLSs score (Fig. 1F, Supplementary Fig. 3D), indicating that secondary tumors may evolve from primary tumors in a similar way to how carcinogenesis arises from normal tissues. However, there was no difference between recurrent and primary tumors in TLS signature expression and score (Supplementary Fig. 3C, D).

### TLSs shaped an inflamed immune infiltrative landscape

**Fig. 2:**
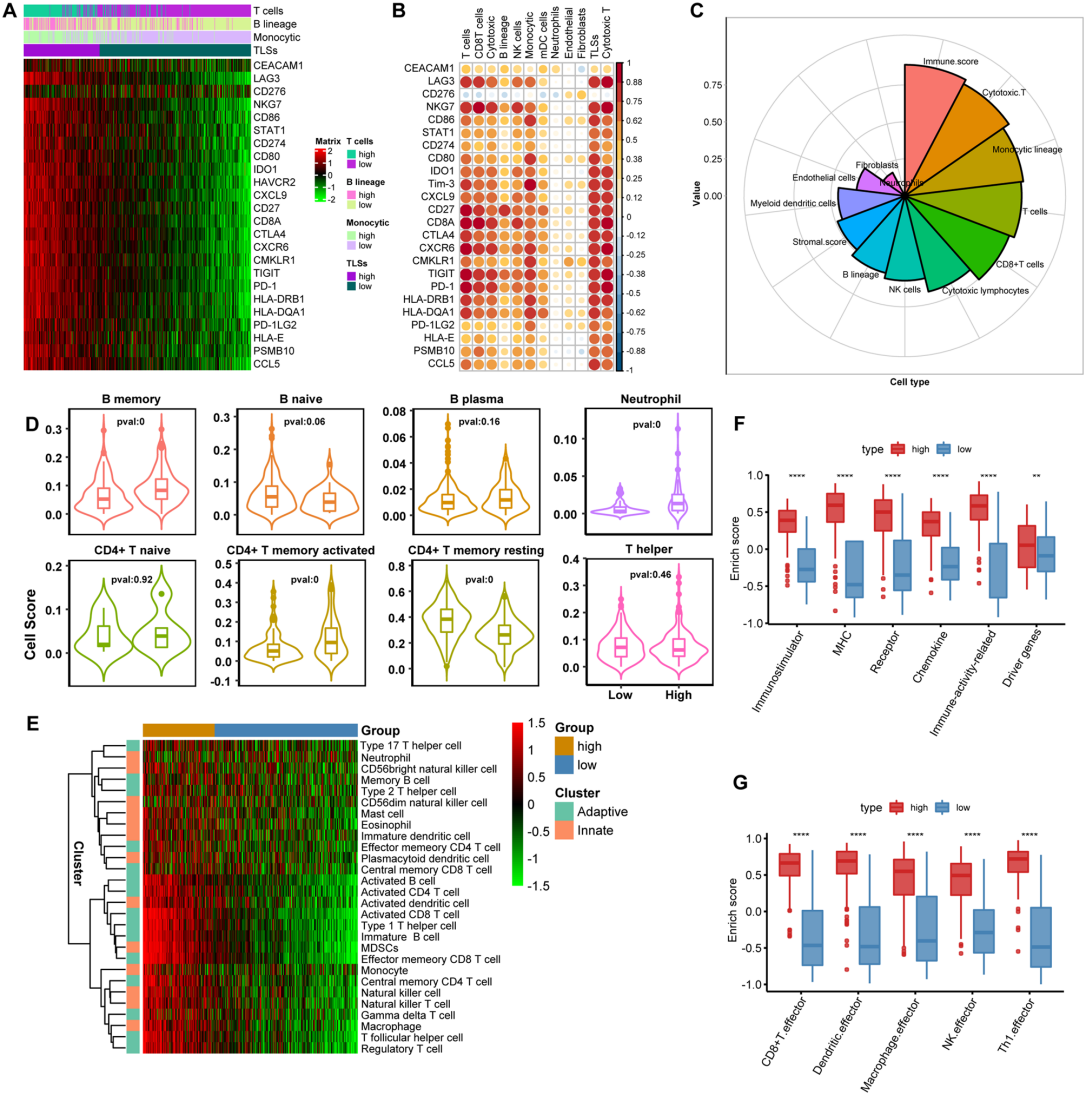
TLSs and immune microenvironment. **A** Expression profile of immune checkpoints according to the stratification of TLSs statue, T cell and B lineage. **B** Correlation analysis of TLSs, immunocytes, stromal cells with immune checkpoints. **C** Relevance analysis of TLSs with immunocytes, immune score, stromal cells and stromal score. **D** Immunocytes infiltration analysis between TLS-hi and TLS-low groups through CIBERSORT. **E** Diversity of 28 tumor-infiltrating immunocytes between TLS-hi and TLS-low groups. **F** Evaluation of immune function presenting as enrich score between TLS-hi and TLS-low samples. **G** Difference of effector gene evaluation of tumor-associated immune cells between high and low TLSs groups (dataset: TCGA-HNSCC)

The presence of tumor-infiltrating TLSs may be associated with increased immunocyte invasion and immunological microenvironment. The majority of immune checkpoints and T cell receptors, including P*D-L1, LAG3, TIGIT, HAVCR2, PD-1, CD80, CTLA4* and *IDO1,* showed higher expression in TLS-hi tumors (Fig. 2A). The expression relevance of these checkpoints was more significant in TLSs than in T cells, CD8 T cells, cytotoxic T, B lineages, NK, monocyte and mDC (Fig. 2B). TLSs were also associated with higher immune score and infiltration of cytotoxic T, monocytic lineage and T cells, but not stromal parameters, which directly indicates good responses to immunotherapy (Fig. 2C). Subsequently, we probed the tumor infiltrative immune landscape between the TLS-hi and TLS-low groups. A higher enrichment of memory, immature and activated B cells, but not naïve and plasma B cells, and increased proportions of memory and activated CD4 + T and CD8 + T cells (*p* < 0.001), and activated dendritic cells, type I T helper cells, MDSC, were found in TLS-hi tumors (Fig. 2D, E, Supplementary Fig. 4A, B). To explore whether tumor subsite poses a strong bias in the immune features between TLS-high and TLS-low phenotypes, we categorized HNSCC into three subtypes, including hypopharynx, oral cavity and oropharynx. Cancers from these tumor subsites showed similar immune cells infiltration and transcript level of immune checkpoints (Supplementary Fig. 5), and from which we can exclude the tumor subsite bias.

In addition, the TLS-hi subtype had a more powerful immunostimulatory profile, high expression of chemokines, a higher enrichment in human leukocyte antigens (MHC) and TCRs, and a higher score of tumor-infiltrating immune cells (TIICs) (CD8 T, dendritic cell, macrophage, NK, Th1) effectors (*p* < 0.0001), demonstrating a better function of these immune cells (Fig. 2F, G).

**Fig. 3:**
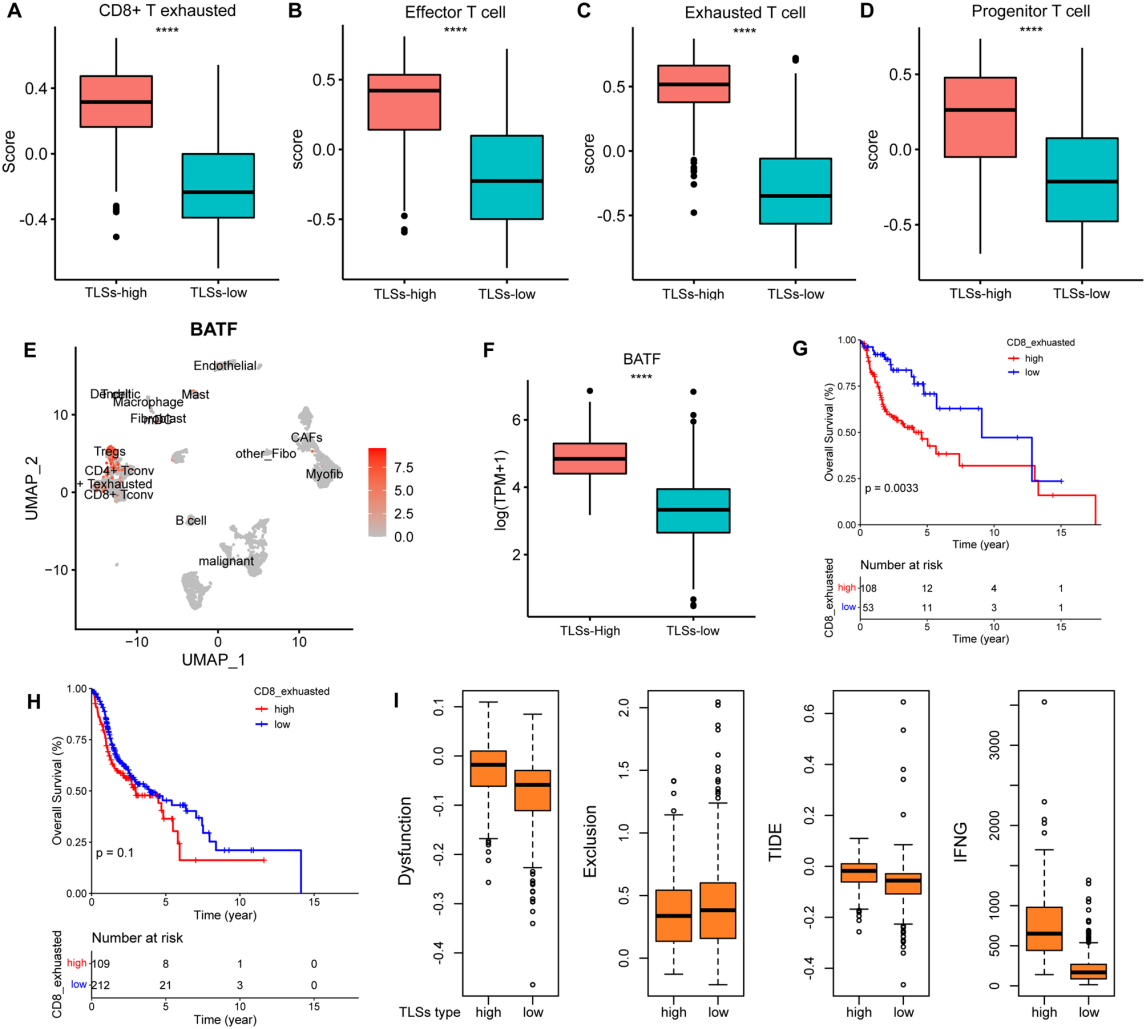
TLSs and exhausted CD8 + T cells. **A** Infiltration of exhausted CD8 + T cells between TLS-hi and TLS-low groups. **B**–**D** Infiltrating score of effectors, exhausted and progenitor T cells between high and low TLSs samples. **E** BATF expression analysis in HNSCC single cell RNA-seq data. **F** BATF expression in HNSCC bulk RNA-seq data between TLS-hi and TLS-low groups. **G**, **H** The determined role of TLSs in exhausted CD8 + T cell related prognosis. **I** T cell dysfunction and exclusion, *IFNγ* analysis between high and low TLSs groups (dataset: TCGA-HNSCC, single cell data, 2017)

The relatively higher infiltration level of exhausted CD8 + T cells in TSL-hi tumors was also found in current analysis (*p* < 0.0001) (Fig. 3A). To further validate this discovery, CD8 + T cells are further classified into three groups: (1) Ly108^+^ progenitor T cells (Tpro), with high capacity to produce IFN-γ and TNF-α, (2) CX _3_ CR1^−^ Ly108^−^ exhausted T cells (Texh) with the lowest potential to produce inflammatory cytokines, represented as truly exhausted phenotype; (3) CX _3_ CR1^+^ cytolytic T cells (Teff) with capacity to expression Granzyme B and cytolytic function, during persistent infection or tumor progression [16]. We demonstrated that all these three subtypes showed obvious elevation in TLSs-hi groups (*p* < 0.0001) (Fig. 3B–D). *BATF*, a key transcription factor during progenitor to cytolytic effector CD8 + T cells, is specifically expressed in Tregs and exhausted CD8 + T cells among HNSCC scRNA-Seq data (Fig. 3E). As expected, *BATF* also highly enriched in TLSs-hi samples as compared to TLSs-low samples (*p* < 0.0001) (Fig. 3F). Exhausted CD8 + T cells hold a negative correlation with immune response and overall survival. We next probed into whether TLSs statue had a decisive role to the clinical significance of exhausted CD8 + T cells. In TLSs-hi group, patients with high infiltration of exhausted CD8 + T cells showed worse outcome (*p* < 0.01) (Fig. 3G). When it comes to TLSs-low group, there is no difference in OS rate between high and low infiltration groups (Fig. 3H). Immune evasion has an intimate correlation with T cell dysfunction and exclusion. An interesting finding was that TLSs-hi group hold more dysfunction T cells, TIDE score and higher concentration of IFN-γ, whereas TLSs-low group had higher exclusion score, indicating that TLSs-hi and TLSs-low patients experience immune scape through different mechanisms (Fig. 3I).

### Crosstalk between TLSs and TME of HNSCC

**Fig. 4:**
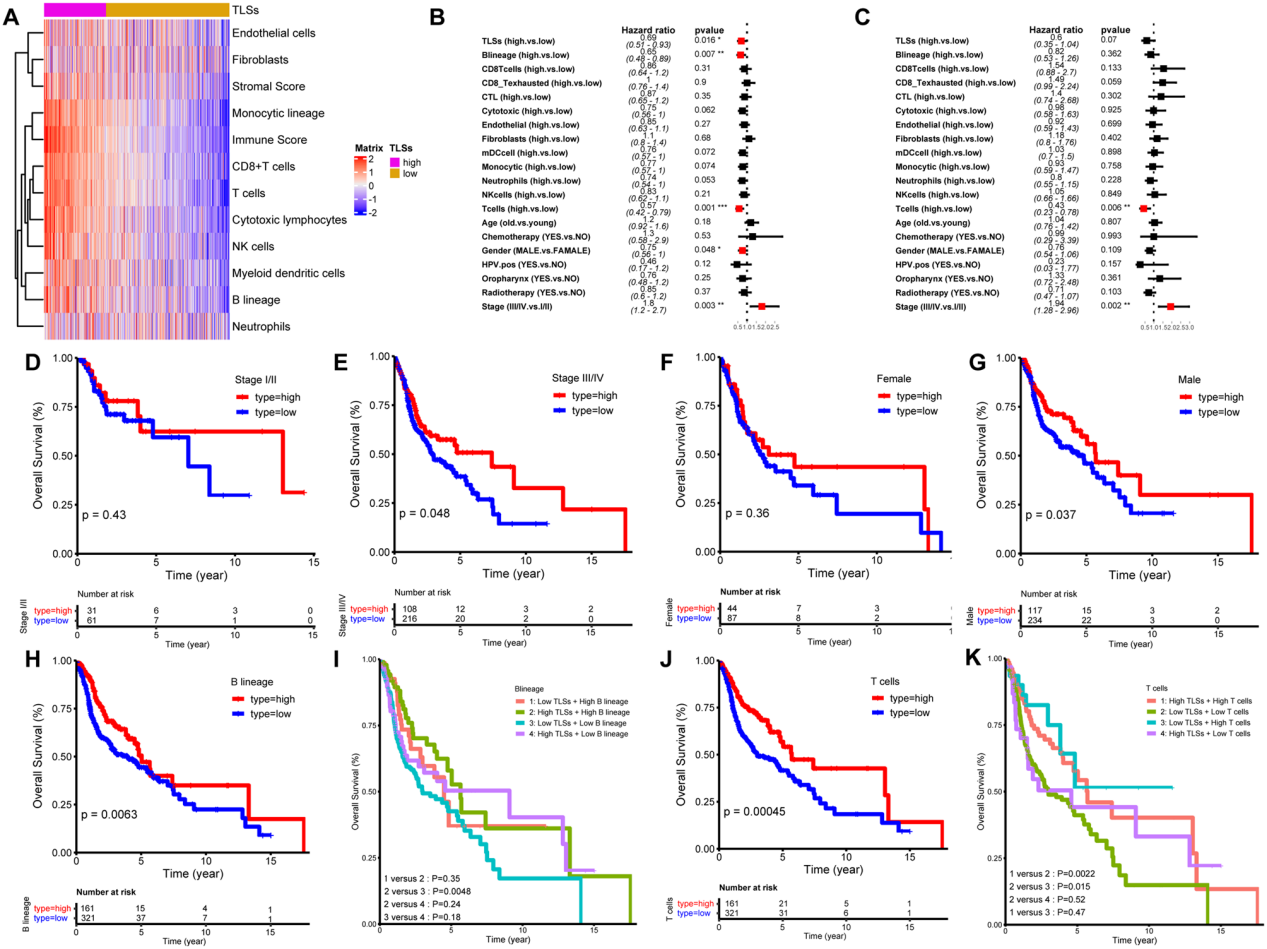
Clinical significance of TLSs. **A** Correlation of TLSs statue with TME. **B** Univariate analysis of TLSs, TME, tumor stage, gender and age. **C** Multivariate analysis of TLSs, TME, tumor stage, gender and age. **D**, **E** The determined role of TLS statue in stage I/II and stage III/IV patients. **F**, **G** The decisive function of TLS statue in female and male patients. **H**, **I** The influence of TLS statue on the clinical significance of B lineage. **J**, **K** The influence of TLS statue on the clinical significance of T cell (dataset: TCGA-HNSCC)

Subsequently, we investigated the interaction between TLS subtypes and the universal landscape of the TME by focusing on immune cells, immune scores, stromal cells and stromal scores. We discovered that TLSs mainly participated in immune reactions (monocytic lineage, immune score, CD8 T cells and B lineage) but showed little correlation with stromal cells and scores, even though stromal cells also expressed these chemokines of TLSs signature (Fig. 4A). We then integrated TLSs, immune-stromal parameters, tumor stage, gender and age as a whole panel for survival assessment. In the univariate analysis, TLSs, B lineage cells, T cells, tumor stage and gender were significantly different, and only T cells low (*p* < 0.01) and tumor stage (III/IV) (*p* < 0.01) were retained as prognostic predictive markers for HNSCC in the multivariate analysis (Fig. 4B, C). As expected, the TLS subtype played a significant role in the clinical outcome of advanced (stage III/IV) HNSCC (*p* < 0.05) but not in that of early-stage HNSCC (Fig. 4D, E). Interestingly, we also found that TLS-hi represented a favorable prognosis in male patients (*p* < 0.05). (Fig. 4F, G).

We then tried to identify the determined role of TLSs on the clinical significance of immunocytes. Higher infiltration levels of T cells and B lineage cells in the TME signified better overall survival (*p* < 0.01) (Fig. 4H and J). However, cytotoxic T cells, neutrophils and mDCs were not predictive for OS outcomes (data not shown). Subsequently, we investigated whether the presence of TLSs has a measurable influence on the clinical significance of T cells and B lineage cells. As shown in Fig. 4I and K, TLS abundance was not associated with the level of B lineage cells both in the high and low groups. Regarding T cells, TLS-hi tumors showed relatively better OS outcomes than TLS-low tumors in the T cell high group but not in the T cell low group (Supplementary Fig. 6A, B). These results indicate that TLSs have a determined role in the clinical significance of T cell levels. To confirm the predictive role of T cell and B lineage in overall survival of HNSCC, we integrated T cell and B lineage with stage, HPV status, oropharynx, gender and age, with the finding that both HPV status and oropharynx have no determined role in the clinical significance of T cell and B lineage (Supplementary Fig. 7A–D).

### Molecular mechanism of TLSs mediated immune landscape remodeling

**Fig. 5:**
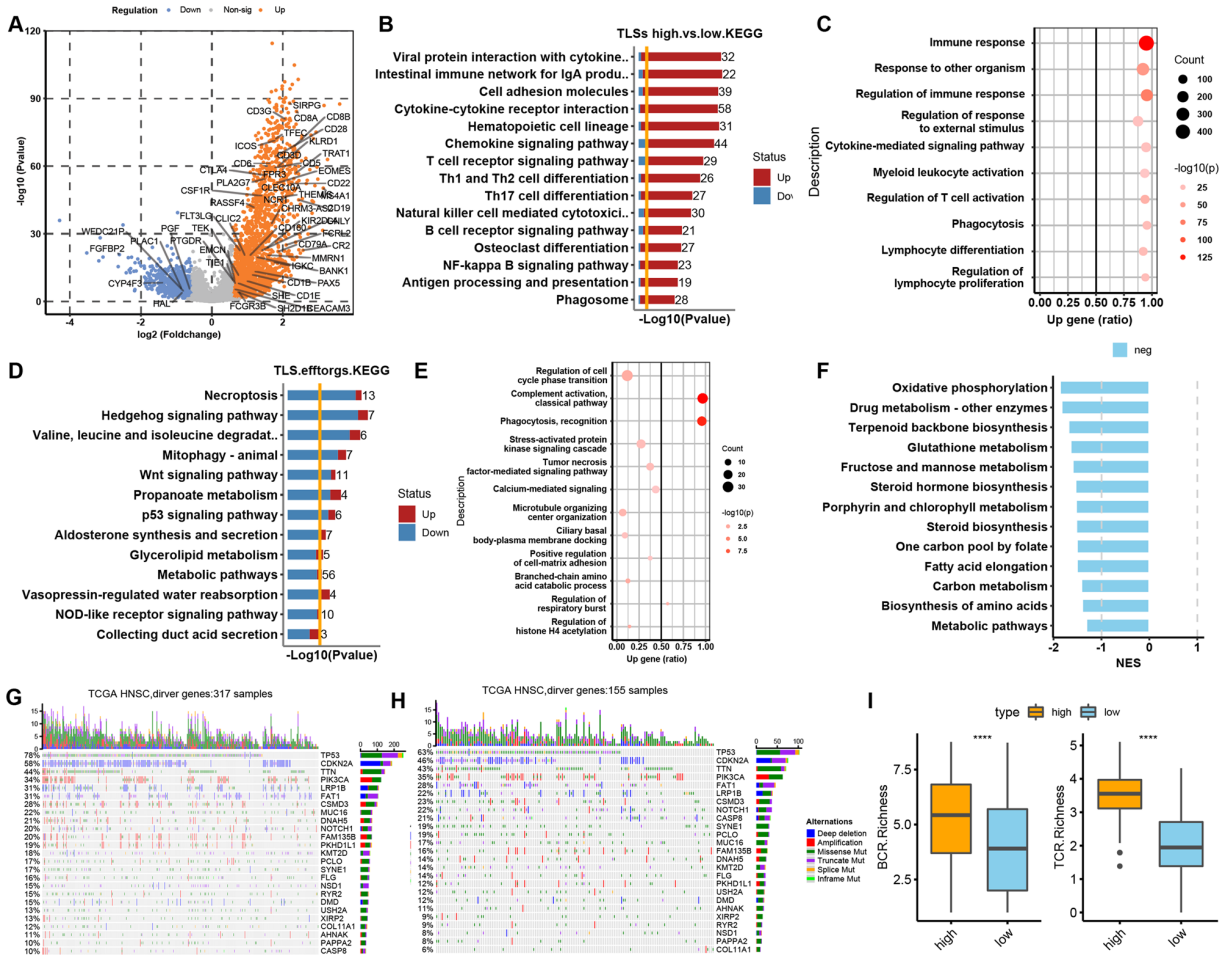
Gene profiling and mutation analysis in different TLS subtypes. **A** Volcano plot of different expression genes (DEG). **B**, **C** KEGG and Gene Ontology (GO) enrichment analysis of DEG between TLS-hi and TLS-low groups. **D**, **E** Cox-PH survival regression to test the level of TLS interacts with other genes in the tumor to affect survival outcome. **F** Metabolic difference between high and low TLSs samples. **G**, **H** Tumor driver gene mutation landscape between two TLS subtypes. **I** BCR and TCR richness between TLS-hi and TLS-low groups (dataset: TCGA-HNSCC)

To unravel the molecular mechanisms and functional changes between TLS-hi and TLS-low groups, differentially expressed genes (DEGs) were identified from the HNSCC TCGA gene set (Fig. 5A). Subsequently, intrinsic pathway alterations were analyzed based on these DEGs, characterized by significant enrichment of cytokine–cytokine receptor interaction, chemokine/TCR/BCR signaling, Th1/2/17 differentiation, NK cell-mediated cytotoxicity, and antigen processing and presentation (Fig. 5B). The bubble chart further confirmed these discoveries and emphasized enrichment in immune response, T cell activation, and lymphocyte differentiation and proliferation (Fig. 5C). We then tried to explore whether TLS subtype associated DEGs have correlation with stromal and immune scores. In HNSCC samples, stromal and immune scores have been categorized as stromal-hi/low and immune-hi/low according to the DEGs (Supplementary Fig. 8 A–C). Then the up- and down-expressed genes were presented in TLSs hi/low, stromal-hi/low and immune-hi/low, respectively (Supplementary Fig. 8 D–F). Finally, DEGs of different subtype of stromal and immune score were merged with that of TLS subtype, with the finding that TLS had more intimate association with immune score than stromal score (Supplementary Fig. 8G).

Subsequently, we investigated the level at which TLSs interact with other genes or signaling pathways in HNSCC tumors to affect the overall survival. We finally filtered 102 genes, among which 63 genes had positive coefficient and the remaining 39 genes hold a negative coefficient. Pathway enrichment showed that necroptosis, mitophagy, *p53* and *Wnt* signaling, and the *NOD*-like receptor signaling pathway, complement activation, tumor necrosis factor-mediated signaling pathway, which served as anticancer effectors, enhanced the association between TLSs and better clinical outcomes, which showed the synergistic effects of TLSs and the enriched signaling pathways in contributing to a more favorable prognosis (Fig5D, E). When the selective threshold *p* value was adjusted to ≤ 0.001, the metabolic and *AMPK* signaling pathways were filtered and determined to be associated with TLSs (Supplementary Fig. 9A). Inspired by these enrichment results, we probed into the metabolic landscape in tumors with different TLS subtypes. As shown in Fig. 5F, TLS-hi tumors were featured by enhanced negative regulation of oxidative phosphorylation, glutathione metabolism, carbon metabolism, glycoprotein biosynthesis and positive regulation of lipid, phospholipid and phosphorus metabolic processes, indicating a metabolic shift from carbohydrate to lipid metabolism in TLS-hi tumors in comparison with TLS-low tumors.

High tumor mutational burden (TMB) was significantly associated with a higher survival rate among HNSCC patients (*p* < 0.05) (Supplementary Fig. 9B). We then probed into the influence of TLS subtype on the TMB in HNSCC. Unfortunately, the TLS subtype has no determined role in the clinical significance of TMB (data not shown). Subsequently, we explored the driver genes and their mutation diversity between the TLS-hi and TLS-low groups. The commonly mutated genes with high frequency across the two groups are listed in Fig. 5G, H, which showed a higher mutation frequency of *MUC16* (22% vs. 17%), *DNAH5* (21% vs. 14%), *PKHD1L1* (19% vs. 12%), *NSD1* (15% vs. 8%), *KYR2* (15% vs. 9%), and *COL11A1* (12% vs. 6%) in TLS-hi versus TLS-low tumors. However, *CASP8* (22% vs. 10%) was found to have a strikingly higher mutation frequency in the TLS-low group. Regarding the mutation types, no obvious difference was found in the number of deletions, in frame and splice mutations (Supplementary Fig. 9C, D). A slight change in amplification mutation frequency and notable diversity in missense and truncating mutations were observed between TLS-hi and TLS-low tumors (Supplementary Fig. 9E). In addition, we also found that TLS-hi tumors hold a higher TCR and BCR richness (*p* < 0.0001) (Fig. 5I). These variations in the mutation landscape and TCR/BCR may contribute to the predictive effect of TLSs on survival outcome.

### The role of innate lymphoid cells and CAFs in TLSs neogenesis

**Fig. 6:**
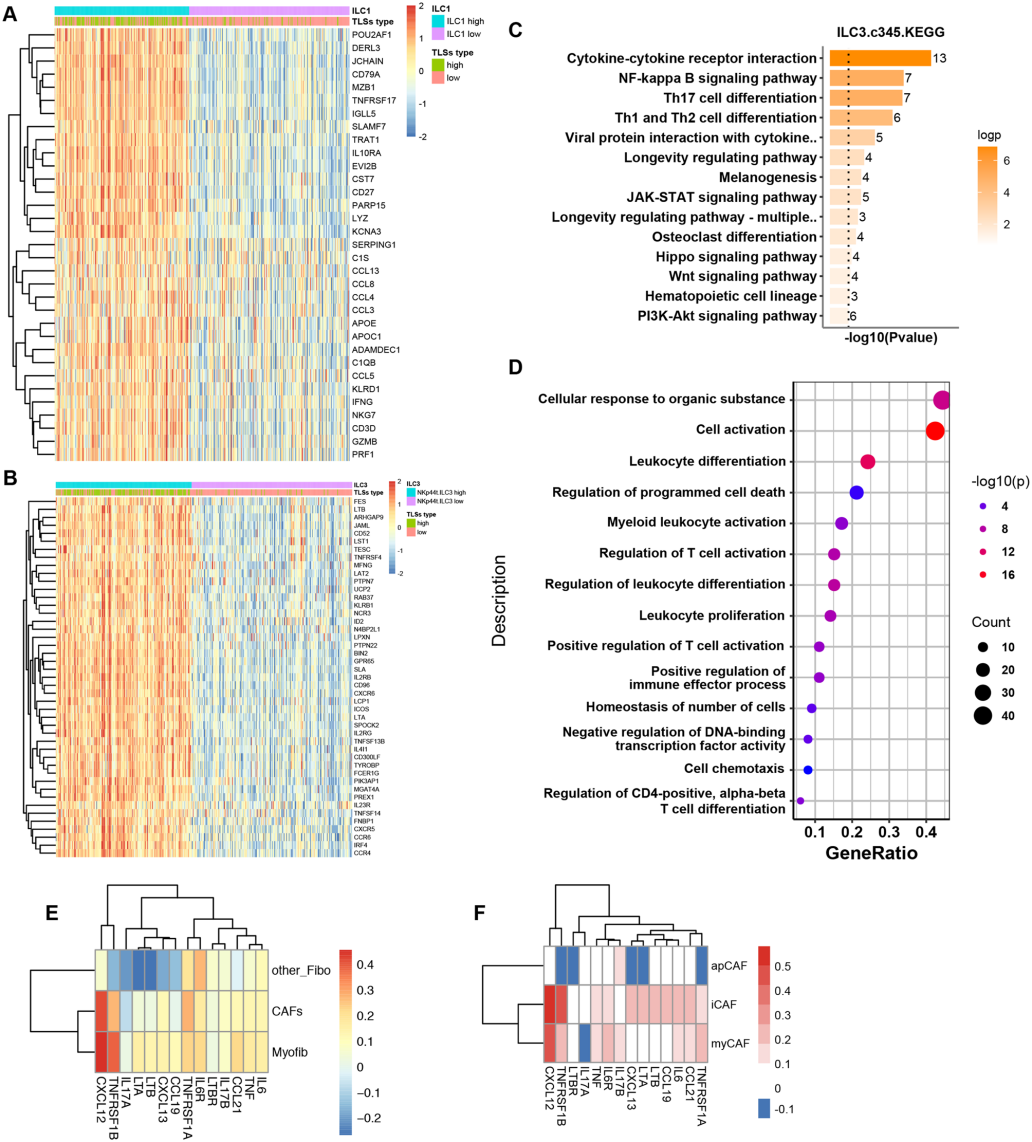
The role of innate immunocyte and fibroblasts in the neogenesis of TLSs. **A** Gene profiling analysis based on the TLSs statue and ILC1 infiltration. **B** Gene profiling analysis according to the stratification of TLSs statue and ILC1 infiltration. **C**, **D** KEGG and GO analysis of DEG between ILC3-hi and ILC3-low samples. **E**, **F** The correlation of different subtype of fibroblasts with gene set associated with TLS formation (dataset: GSE137564)

Innate lymphoid cells (ILCs) are tissue-resident lymphocytes composed of group 1 LCs (ILC1), including NK cells and non-NK cells, group 2 LCs (ILC2) and group 3 ILCs (ILC3), containing ILC3 cells and lymphoid tissue inducer cells (LTi), and participate in the TLS induction. In HNSCC samples, the signature of ILC1 and ILC3 was co-expressed at a high level with that of TLS-hi subtype, demonstrating that the presence of ILC1/3 cells may have a positive correlation with TLSs induction (Fig. 6A, B). More specifically, co-expression analysis of TLSs and ILC3 signatures indicated an enrichment of the *LTα, LTβ* and *TNF* families, including *TNFRSF4, TNFSF13B* and *TNFSF14*, which have an intimate association with TLS neogenesis (Fig. 6B). Further pathway enrichment confirmed the biological function of ILC3 cells featured by the activation of Th1/2/17 differentiation, cytokine–cytokine receptor interaction, positive regulation of immune effector process, programmed cell death and T cell activation (Fig. 6C, D). In regard to ILC1 cells, significant elevation of chemokine, Toll-like receptor signaling pathway, NK cell-mediated cytotoxicity and immune response were observed, implying that ILC1 cells and ILC3 cells exert distinct functions during TLS neogenesis and further immune response (Supplementary Fig. 10A, B).

Besides innate immunity, stromal cells, such as fibroblasts, have also been reported to participated in TLS neogenesis. To clarify which subtype of fibroblasts contributing to the TLS development, we collected the pivotal genes mediating the TLSs neogenesis, such as *IL-6, TNF-α, LTA, LTB, CCL19, CCL21, CXCL12, CXCL13, TNFRSF1A/B* and *IL-17*. Then we calculated the correlation of these marker genes with the fibroblasts from HNSCC single cell data and identified that cancer-associated fibroblasts (CAFs) and myofibroblast hold the positive association with TLSs neogenesis (Fig. 6E). Furthermore, inflammatory CAF (iCAF) and myofibroblast CAF (myCAF) showed the most relevant with this gene set as compared with antigen-presenting CAF (apCAF), demonstrating that these two subtypes of CAFs may have an important role in TLS neogenesis (Fig. 6F).

### Novel HNSCC subtypes based on TLS statue and TME parameters

**Fig. 7:**
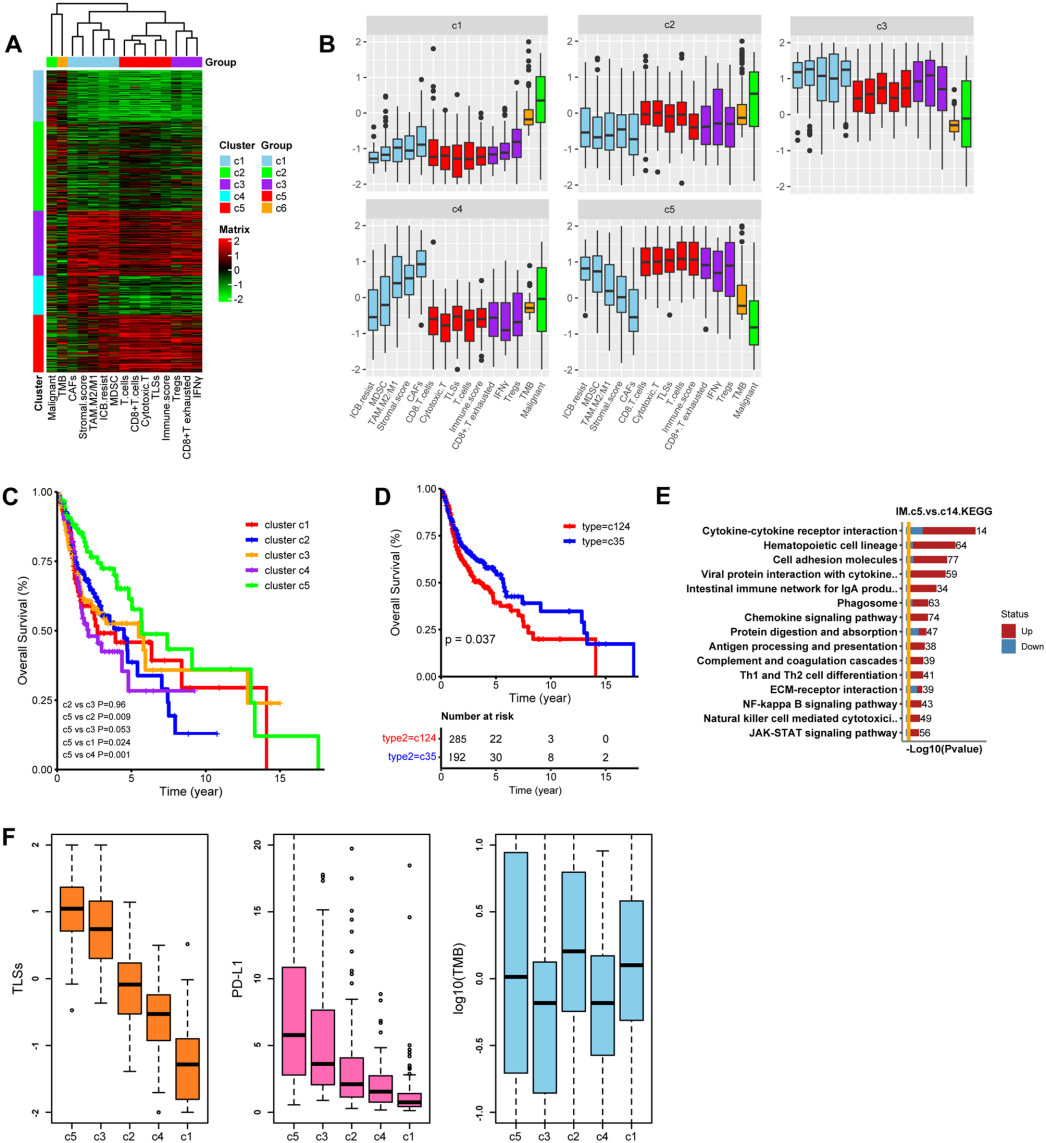
Novel TME classification of HNSCC. **A** Unsupervised clustering of TME parameters, including immunocytes, immune score, stromal cells and score, TLSs, *IFNγ*, TMB and malignant cells. **B** Relative value distribution of TME parameters among c1, 2, 3, 4 and 5. **C**, **D** Overall survival analysis of patients within different clusters. **E** KEGG analysis of DEG between cluster 5 and cluster 1, 4. **F** The distribution of TLSs score, *PD-L1* and TMB among different clusters (dataset: TCGA-HNSCC)

We next proposed a novel HNSCC TME classifications (HNSCC-TCs) based on the comprehensive estimation of the whole tumor landscape, including stromal cells and stromal score, TLSs, T cell dysfunction and immune evasion features, antitumor effective cells and malignant tumor cells (Fig. 7A). Five HNSCC-TCs presented the high heterogeneous TME profiles. HNSCC-TC 1, the “immune desert” subtype, scarce infiltration of immune-promoting and suppressive cells, low level of TMB and TLSs, and high proportion of malignant cells. HNSCC-TC 2, a few infiltrations of both immune cell subtypes, high level of TMB and low level of TLSs, and high proportion of malignant cells. HNSCC-TC 3 features high infiltration level of both types of immune cells, low level of TMB and high level of TLSs, and a high density of stromal cells and high stromal score. HNSCC-TC 4 features scarce infiltration of immune cells, a high density of stromal cells and high stromal score, low level of TMB and TLSs. HNSCC-TC 5, high infiltration of both types of immune cells, a moderate density of stromal cells and stromal score, and high level of TMB and TLSs (Fig. 7B). Subsequently, we verified the feasibility and rationality of the current classification by association analysis with patient survival outcomes. Patients with HNSCC-TC 4 exhibited the worst OS outcomes in comparison with those with HNSCC-TC 1, indicating that a high density of stromal cells aggravated the immune desert environment and exerted synergistic effects on immune evasion and tumor progression. This finding was also proven by the OS rate difference between HNSCC-TC 3 and HNSCC-TC 5 patients, which was also characterized by increased infiltration of stromal cells and a higher stromal score. The prognosis of HNSCC-TC 2 was comparable with that of HNSCC-TC 3, signifying that the higher infiltration of immunologically promoted cells could be counteracted by the abundance of stromal cells and other immunosuppressive factors. Patients with HNSCC-TC 5 showed the best OS prognosis as compared to other groups, featuring with high infiltration of immune cells and high score of TLSs, low score of stromal (Fig. 7C). In addition, a positive correlation of enriched stromal elements with low TMB, such as in HNSCC-TCs 3 and 4, indicated that stromal factor is also an influencing parameter for determining immunotherapy responses and patient prognoses (*p* < 0.05) (Fig. 7D).

We then explored the TLS distribution in HNSCC-TC 1–5 and found the highest level of TLSs in HNSCC-TC 5, followed by HNSCC-TC 3, 2, 4, and 1, showing identical trends with PD-L1 expression but not TMB distribution, which further confirmed the co-expression of TLSs and immune checkpoints (Fig. 7F). As for immune evasion, HNSCC-TC 3 presented higher T cell dysfunction score than HNSCC-TC 5, and HNSCC-TC 4 was estimated with higher exclusion score, signifying that stromal score play a determined role in T cell dysfunction and exclusion, thus promoting the immune scape (Supplementary Fig. 10C). This TME classification in HNSCC TCGA was re-verified in another database and showed good repeatability in cluster distinguish and survival prediction (Supplementary Fig. 11A). The difference of overall survival among different clusters were similar to that of TCGA-HNSCC (Supplementary Fig. 11B). Furthermore, we discussed the dynamic change of TLS level during ICB treatment with the findings that TLS level showed no significant difference between CR/PR and SD groups, but revealed higher TLS enrichment in CR/PR patients than PD patients (Supplementary Fig. 11C).

We then probed into the underlying mechanism contributing to the difference in TME composition between HNSCC-TC 5 (the subtype with the most abundant immune-promoting factor and the best prognosis) and HNSCC-TC 1 and 4 (the subtype with a scarcity of immune-promoting factors and the worst prognosis). A significant reduction in cytokine-cytokine interaction, chemokine signaling, antigen processing and presentation, and T cell differentiation, was observed in the HNSCC-TC 1 and 4 groups, which indicated a lower immune response activity and stronger stromal biological process (Fig. 7E). We also performed further in-depth analyses to identify markers that were specifically expressed in tumor cells and found that 9 genes (*COL4A6, LANCL2, PLA2R1, PLAC1, IL1A, MAGEA12, RNF128, GLI2* and *DMP1*) participated in the inhibition of TLS neogenesis, which deserved further molecular investigation.

### The prediction role of TLSs in immunotherapy

**Fig. 8:**
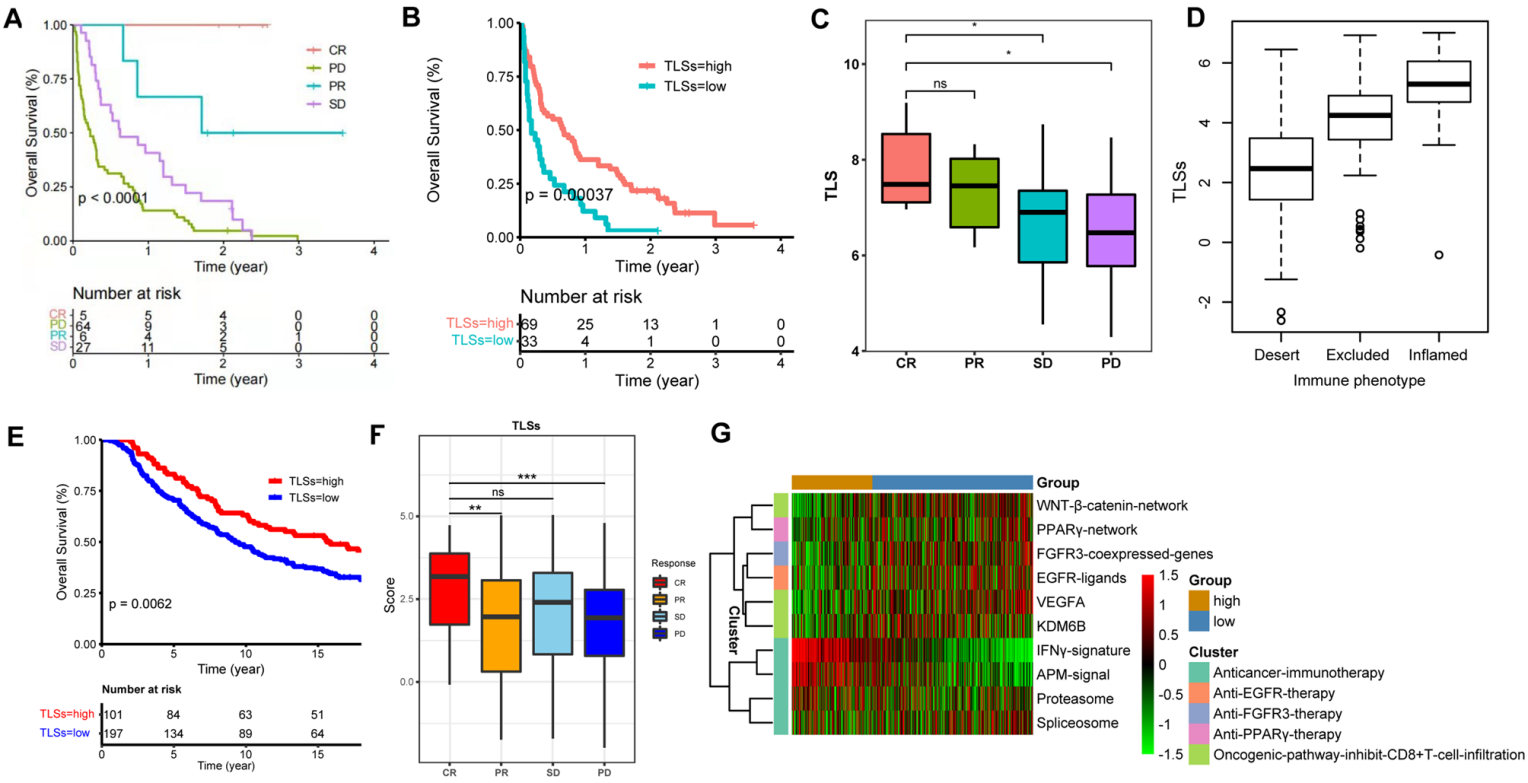
Prediction role of TLSs in immunotherapy. **A** Overall survival analysis among CR, PR, PD and SD patients. **B** The influence of TLS statue on the overall survival of HNSCC-ICB treatment cohort. **C** TLSs distribution among CR, PR, PD and SD patients. **D** TLSs distribution among desert, excluded and inflamed immune phenotype. **E** The influence of TLS statue on the overall survival of urothelial carcinoma-ICB treatment cohort. **F** TLSs distribution among CR, PR, PD and SD patients. **G** KEGG analysis of DEG between TLS-hi and TLS-low groups in ICB treatment cohort (dataset: IMvigor210)

Finally, we probed whether TLS subtypes are intimately associated with different therapeutic regimens. Unsurprisingly, TLS abundance was not associated with the regulation of radiotherapy, chemotherapy or *EGFR* targeted therapy processes (Supplementary Fig. 12). We then investigated whether the established TLS signature plays a role in OS outcome and immunotherapy response prediction. In HNSCC- immunotherapy cohort (GSE159067), a total of 102 patients were treated with PD-1/PD-L1inhibitors, and TLS status also showed its predicative role in overall survival of HNSCC with a *p* value of 0.028, and the TLS score was highest in the CR patients, followed by the PR, SD/PD patients (Fig. 8A–C). As expected, TLS level presented a gradually increased trend among desert, excluded and inflamed immune phenotypes (Fig. 8D).

Subsequently, we verified the signature using urothelial carcinoma data from GSEA, which containing 348 patients who received Atezolizuma, a PD-L1 antibody. In this ICB-treated cohort, patients were categorized into two groups according to the PD-L1 expression in tumor biopsies, IC1(1 <  = PDL1 < 5%) and IC2/3 (PDL1 > 5%), with the finding that IC2/3 patients reached objective response rate (ORR) at 26% and CR rate at 11%, and a higher OS rate in CR/PR patients as compared with that of SD/PD patients. Data analysis presented that patients with high TLS levels hold significantly better OS rates than those with low TLS levels (*p* < 0.01) (Fig. 8E). The CR group had the highest abundance of TLSs than that in the PR (*p* < 0.05) and PD groups (*p* < 0.001), whereas there was no significant difference in TLSs level between the CR and SD groups (Fig. 8F). What’s more, we verified the expression of different gene sets represented various treatments (not performed in HNSCC-immunotherapy cohort due to the lack of transcript data of relative genes), such as immunotherapy, anti-*EGFR* therapy, anti-*FGFR3* therapy and anti-*PPARγ* therapy, and found that TLSs was intimately correlated only with immunotherapy (Fig. 8G).

## Discussion

TLSs are lymphoid neogenesis that occur in nonlymphoid tissues, such as TME, under chronic inflammation and immune stimulation [17]. Currently, TLS detection is mainly performed by IHC analysis of pathological sections, which is valuable for definitively determining the presence of TLSs. However, there are several limitations of IHC-based TLS section analysis, such as the consumption of excessive clinical sample resources, inconvenience in quantitative analysis, complexity in combination analysis with TME, and difficulty in performing underlying mechanism elucidation [9, 10]. Therefore, there is a robust demand for the establishment of TLS gene signatures using transcriptomic sequencing data, which will be more suitable for TLS-related clinical and basic researches.

Previously, scholars have attempted to identify TLS neogenesis and TLS presence-related biomarkers. First, *CCL19, CCL21, CXCL12*, and *CXCL13* were defined as the main chemokine genes associated with TLSs [18]. TLSs are composed of mature DCs, the T cell zone and the B lineage zone, indicating the occurrence of lymphoid neogenesis as the secondary lymphoid organs adapted to inflammatory signals [10]. Based on the cellular composition in TLSs, a 12-chemokine signature *(CCL2, CCL3, CCL4, CCL5, CCL8, CCL18, CCL19, CCL21, CXCL9, CXCL10, CXCL11, CXCL13)* and 9 gene signature *(CCL19, CCL21, CXCL13, CCR7, SELL, LAMP3, CXCR4, CD86, BCL6)* were proposed with the conclusion that this signature showed an extremely heterogeneous distribution among different cancer types [13, 14]. More specifically, some tumor types, such as lung adenocarcinoma, squamous cell carcinoma, glioblastoma and uveal melanoma, presented with high level of TLSs. However, other malignant tumors, including adrenocortical carcinoma, paraganglioma and pancreatic cancers, showed a relatively low proportion of TLSs [10]. These findings suggested that the TLS signature should be applied to the specific TME due to the high heterogeneity. We integrated chemokines reported by other cancer types and performed screening by clustering classification and eliminating the genes with low correlation and established a 13-gene signature for HNSCC. Through prognostic analysis and ICB therapy verification, we proved the rationality of the current TLS 13-gene signature in HNSCC.

The presence of TLSs was associated with a lower risk of early recurrence of hepatocellular carcinoma [9, 19], TNM stage refinement in non-small cell lung cancer [20], immunological strategies for triple‑negative breast cancers [21]and upper tract urothelial carcinoma [22] and was also found to be an innovative prognostic biomarker for HNSCC [5, 12]. What’s more, TLSs mainly participate in adaptive immune responses and could be deemed as prognostic and predictive factors. To expound the correlation between TLSs and immunotherapeutic responses, clinical trial-related ICB-treated cohorts, including primary and metastatic melanoma (NCT02519322, NCT02437279), renal cell carcinoma (NCT02210117) and soft tissue sarcoma (SARCO28) cohorts, have been investigated and revealed that B lineage cell-enriched TLSs are ideal biomarker for ICB therapy efficacy prediction and are valuable for precise clinical decision making [6–8]. In a cohort of breast cancer with 1058 patients, the high density of both T and B lymphocytes within TLSs in pretreatment biopsy samples was positively correlated with pCR following neoadjuvant immune therapy [23]. This predictive role of TLSs on the immune response may be ascribed to the TME modulation induced by TLSs neogenesis. However, the influence and underlying mechanism of TLS neogenesis on the TME modulation, especially the immune cell composition and immune responses, have been preliminarily deciphered. The presence of TLSs may signify that tumor antigens are recognized by the antigen-presenting cells and further T lymphocytes. TLS-hi tumors are characterized by increased infiltration of activated T cells, memory-type CD8 + T cells and T_H_17 cells with enhanced chemotaxis and cytotoxicity, and decreased proportion of immunosuppressive cells [24]. TLSs in tumors also hold the capacity in the education of intratumoural T cells into effector and memory statues, which verifies that TLSs support the activation of CD8 T cells and further attacking against tumor cells, resulting in the best survival outcome in tumors with the presence of both TLSs and CD8 T cells. Conversely, CD8 T cells in tumors without TLSs infiltration showed high expression level of TIM-3 and PD-1, indicating that those CD8 T cells were more prone to develop into exhausted statue and dysfunctional molecular phenotype, which failed to response to ICB [6].

Few studies have focused on the association analysis of TLSs with T cell exhaustion in the TME. In line with the findings in triple-negative and *HER2* + subtypes of breast cancer, a higher proportion of exhaustive T cells was also found in HNSCC samples [25]. Reviewing the HNSCC data, we found that a high density of T cells or CD8 + T cells may contribute to the higher proportion of exhausted T cells. The exhausted phenotype of T cells could be deemed as a special stage or status of T cells; thus, it is not difficult to understand the higher infiltration rate of exhausted T cells in the immune inflamed microenvironment. In addition, the remaining or surviving malignant tumors in this high immune-infiltrated TME may be more able to induce T cells to transform into exhausted statue.

In addition, TLSs enrichment was positively associated with an augment of intratumor CD4 T cells clonality, characterized by higher infiltration of naïve, central-memory, and activated CD4 T cells and lower distribution of regulatory T cells, which indicated that TLSs neogenesis may be able to narrow the deleterious impact of high Treg density on immune response [26]. In HNSCC, we found a lower abundance of memory-type resting CD4 + T cells, higher infiltration of memory-type activated CD4 + T cells, and no distinct difference in Tregs and T helper cells. The CD20 + B lineage is a representative cell within TLSs. Memory-type but not naïve and plasma-type B cells significantly accumulated in TLS-hi tumors. TLSs are a privileged area responsible for the generation of effector memory cytotoxic cells and memory B cells, thus sustaining a long-term immune response against tumor antigens and promoting further antibody production [27]. In single-cell analysis, uniform elevation of MHC-I and MHC-II in B cells indicated that B lymphocytes within TLSs are capable of antigen presentation. B cells enriched tumors contained more CD 4 and CD8 T cells with naïve and memory features, and CD20 + B cells colocalized with CD4/8 T cells in TLSs of tumors of ICB responders, such synergistic effect of B cells and T cells promote the ICB therapeutic efficacy [6, 7]. Besides T and B cells, TLS-associated mature DCs yield a specific immune compound featured by cytotoxic orientation, thus shaping the immunophenotype of TME and promoting a protective immune response regulated by T cells against cancer [24]. In HNSCC, a higher proportion of activated DCs was also detected in TLS-hi tumors, indicating that TLSs may favor DC priming and activation, which further elicits the antitumoral response of CD8 + T cells [28, 29]. What’s more, TLSs also favor surrounding high endothelial venules (HEVs) in lymphocyte recruitment, and the combination of antiangiogenic and anti-PDL1 therapies increased HEV formation, thus further improving antitumor immune responses [10].

Vaccine trials also illustrated that TLSs may be a major component in the induction of antitumor immunity. Therapeutic vaccination of cancer patients induced the TLSs neogenesis and proliferating lymphocytes in the stroma adjacent to the tumor region, which were not found in non-vaccinated patients. Expression gene and pathway analysis of micro-dissected TLSs showed upregulation of immune cell activation and trafficking, Th17 cell stimulation, and suppressed function of Treg cells [10]. These discoveries demonstrated the potential role of TLSs in converting non-immunogenic TME into immune inflamed tumor ecosystem.

The classical immune classification of the TME is “immune infiltrated,” “immune excluded” and “immune desert,” which was proposed on the basis of the infiltration levels and distribution of CD8 + T cells and other immunosuppressive cells [8]. With the development of bioinformatic techniques, an increasing number of classifications have been proposed, including molecular subtypes based on the gene signature, the combination of immune features with epigenetic information, and the immune cell infiltration landscape [30, 31]. In soft tissue sarcoma, TME compositions were integrated with TLSs to form the “SICs” classification: A, immune desert; C, vascularized; E, immune and TLS high; B and D, immune-low and high profiles. However, only SIC E was associated with TLS status [8]. Due to the high heterogeneity of tumors, this classification cannot be used for other types of cancers. Here, we integrated immunological parameters, stromal cells and score, TLSs, TMB and malignant cells to establish a novel TME classification for HNSCC, through which we proved that infiltrated stromal cells may counteract the high proportions of immune-promoted cells. It is not difficult to understand why the worst survival rate was observed in HNSCC-TC 4 patients as this subtype is characterized by an immune desert and a high density of stromal cells. The co-existence of immune-promoted and immunosuppressive cells, high infiltration of T cells and exhausted T cells, Tregs cells is also of importance. We also found an interesting phenomenon that no significant difference in TLSs level exists between the CR and SD groups. This diversity in therapeutic efficacy may be associated with different prognoses between HNSCC-TCs 3 and 5, which were characterized by identically high levels of TLSs but different stromal scores, indicating that PD tumors may have a higher density of stromal cells, which may facilitate immune evasion.

In conclusion, we established a gene signature containing 13 chemokines to identify the TLS level in HNSCC samples, and then explored the correlation of TLSs with immune check point and immune landscape. Based on these, we further confirmed the clinical significance of TLSs, especially for T cell and B cell. Through gene profiling analysis, we uncovered the underlying mechanism of TLSs in remodeling the HNSCC TME, and preliminarily discussed the potential role of innate immune cells and CAFs in the neogenesis of TLSs, which may provide a pivotal guide for TME intervention. What’s more, combining the immune cells and score, stromal cells and score, TLSs and malignant cells, we established a novel classification of HNSCC. Finally, the predictive role of TLSs in immunotherapy has been verified in ICB-treated cohorts. Further consideration should be paid attention to the verification of TLSs signature in prospective cohort studies with regard to ICB treatment.

## Materials and methods

### HNSCC dataset retrieval and reprocessing

Publicly available HNSCC datasets were obtained from the TCGA (HNSCC), GEO (GSE41613, GSE42743 and E-MTAB-1328, GSE137564, GSE68858, GSE159067), CPTAC-HNSCC and IMvigor210 databases, which contain clinical characteristic, therapeutic regimen, corresponding response, follow-up, RNA-Seq and somatic mutation data. The HNSCC RNA sequencing data (FPKM values) were downloaded via the R package TCGA biolinks and were subsequently transformed into transcripts per kilobase million (TPM), which was identical to the microarray data downloaded from the Array Express database. The somatic mutation data were retrieved, and TMB was calculated by using VarScan2. The raw data from GEO databases were normalized based on the chip platform. A total 482 HNSCC samples in TCGA data were enrolled for overall survival analysis. Due to the data missing of follow-up in 121 patients, 361 patients were finally filtered for DFS, RFS and MFS estimation. TMB data was available in 477 patients. This project was approved by the ethics committee of Shanghai Ninth People’s Hospital Affiliated to Shanghai Jiao Tong University.

### Gene expression profiling, TLS signature and scoring

We retrieved the standard data after log2 scale and RMA normalization from the TCGA database. Then, the geometric mean of the genes (candidates for the TLS signature) in every sample was assessed by an MCP counter method to filter specific markers (*CCL2, CCL3, CCL4, CCL5, CCL18, CCL19, CCL21, CXCL9, CXCL10, CXCL11, CXCL13, CXCR4, LAMP3*) for HNSCC and to form the TLS score. Samples with scores greater than the third quartile were classified as TLS-hi, and those with scores lower than the third quartile were defined as TLS-low [9].

### DEGs, GO, KEGG, GSEA analysis for different TLS subtypes

Differentially expressed genes between TLS-hi and TLS-low cohorts were determined by employing the R package limma and setting the cutoff of statistical significance at a *p* value < 0.05 and an absolute fold-change > 1.5. These up- or downregulated genes were then subjected to Gene Ontology (GO) and Kyoto Encyclopedia of Genes and Genomes (KEGG) analyses using the R package clusterProfiler (v3.14.3) based on the criteria for a cutoff *p* value of < 0.05 and an adjusted *p* value of < 0.2. Those GO categories FDR < 0.05 were considered as significant enriched. While pathways with a *p* value < 0.05 were regarded as enriched. Only those GO categories or pathways contains≧5 DEGs were kept for further analysis.

### Cox-PH survival regression

We applied Cox-PH survival regression to analyze how the CTLs interact with the other genes in the tumor to affect survival outcomes. We used a linear hazard model (= *a* × CTL + *b* × *V* + *d* × CTL × *V* + *c*) with the Cox-PH regression model [32]. In the Cox-PH model, the death hazard was estimated through patient survival information. The variable *V* represents the expression level of a candidate gene in the test. Since we selected datasets where CTL levels correlated with favorable survival outcomes, the coefficient *a* was always negative. The association slope between CTLs and hazard was *a* + *d* × *V*. If the coefficient *d* was positive, a higher *V* level would flatten the slope between CTLs and hazard, indicating a reduced association between the cytotoxic T cell level and better survival outcomes. If *d* was negative, a higher *V* level would sharpen the slope between CTLs and hazard, indicating an increased association between the cytotoxic T cell level and better survival outcomes. Collectively, a positive coefficient “d” indicates a reduced association between the TLS level and better survival outcomes, whereas a negative coefficient “d” signifies an enhanced correlation between the TLS level and better OS outcomes.

### Immunological characteristics of the TME

The immunological features of the TME were composed of immunomodulators, chemokine-chemokine interactions, tumor-infiltrating lymphocytes (TILs), immune checkpoint expression and ICB resistance. First, a panel of 122 immunomodulators containing immune stimulators, MHCs, receptors and chemokines was collected and analyzed as previously reported [33]. Second, the infiltration status of distinct immune cells in the TME of HNSCC between the TLS-hi and TLS-low groups was quantified by using four independent algorithms, including the “CIBERSORT” R package, MCP counter, TIMER and XCELL, which were all analyzed based on the LM22 signature. The calculation results were then remodeled by scRNA sequencing data through LM22. C2 analysis[15]. Third, a signature of 20 immune checkpoints was retrieved to investigate the association between TLSs and the expression of immune repressive molecules [34]. Finally, “ESTIMATE” was further applied to calculate the immune and stromal scores for HNSCC individuals by calculating the single-sample gene-set enrichment analysis (ssGSEA).

### Unsupervised clustering for TME of HNSCC

Unsupervised clustering was applied to clarify different associated patterns of immune and stromal factors. Consensus parameters, such as cancer-associated fibroblasts (CAFs), other stromal cells, stromal score, tumor-associated macrophages (TAMs-M2/M1), ICB resistance, MDCSs, Tregs, exhausted CD8 + T cells, T cells/CD8 + T cells/cytotoxic T cells, TLS, immune score, IFNγ, total TMB and malignant cells, which represent the whole TME landscape, were extracted from the TCGA database and then integrated and clustered according to their intrinsic interactions. Gene signatures related to T cell dysfunction and tumor immune evasion used for clustering were identified from tumor cohorts through association analysis of each marker with TIL infiltration in influencing patient survival after ICB therapy [32].

### Somatic mutation analysis

The somatic mutation datasets of patients in the TLS-hi and TLS-low groups were extracted from the TCGA data portal (https://www.cancer.gov/tcga/). The total number of nonsynonymous mutations was selected as the representative to calculate the total mutational burden of the tumors. The mutation frequency and somatic alterations of common driver genes were listed in both groups. The driver genes of HNSC were identified using the “maftool” R package.

### Statistical analysis

Hierarchical clustering analysis was performed on the R “hclust” function using the complete method to identify the number of clusters in TCGA-HNSC based on the expression pattern of signatures. Univariate and multivariate Cox proportional hazards regression models were used to assess the association between the signatures and overall survival with/without clinical variables. The hazard ratio (HR) and 95% confidence interval (CI) were calculated. One-tailed or two-tailed Wilcoxon rank-sum or student tests were used to compare two groups. The Kaplan–Meier method and log-rank test were conducted to compare survival differences between two tumor groups. All statistical analysis was performed using R/ Bioconductor (version 3.6.1).

### Supplementary Information

Below is the link to the electronic supplementary material.

Supplementary file1 (DOCX 26654 KB)

## Data Availability

The datasets generated during the current study are available from the corresponding author on reasonable request.
