# Supplementary material for: Intratumoral tertiary lymphoid structures promote patient survival and immunotherapy response in head neck squamous cell carcinoma
Source: Cancer Immunol Immunother. 2022 Dec 8;72(6):1505–21. doi: 10.1007/s00262-022-03310-5 (PMC10198854; doi:10.1007/s00262-022-03310-5)
Supplement: Supplementary file 1 — Supplementary file1 (DOCX 26654 KB) [file 262_2022_3310_MOESM1_ESM.docx]

**Supplementary Figures**

**
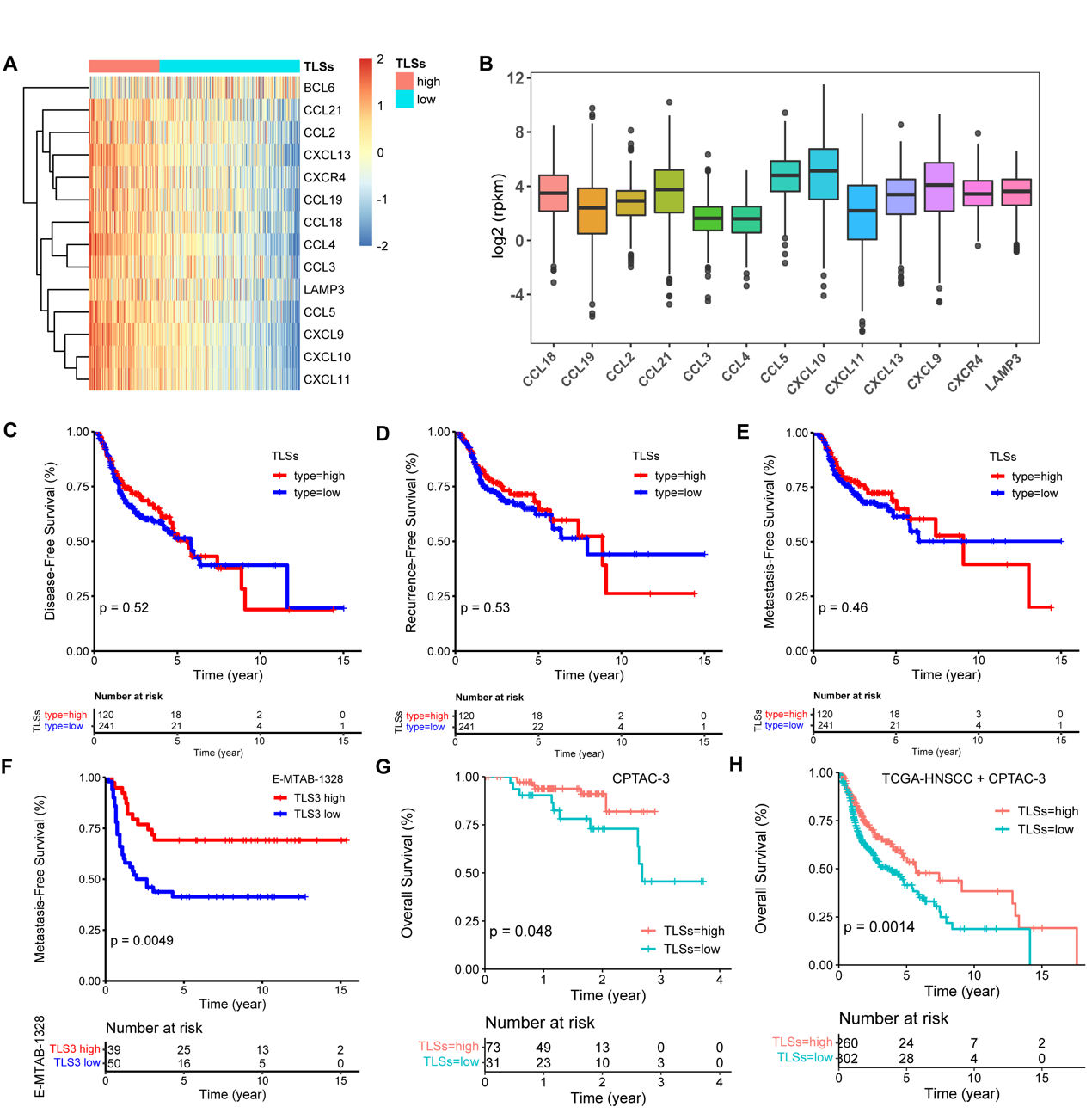
**

**Figure S1.** TLSs signature establishment and clinical prognosis analysis. (A) 14 chemokine expression between TLS-hi and TLS-low groups, from which results BCL6 was screened out. (B) Expression profiling of selected 13 chemokines. (C-E) Disease-free, recurrence-free and metastasis-free survival analysis between high and low TLSs samples. (F-H) The verification of TLSs with HNSCC prognosis in other datasets. (Dataset: TCGA-HNSCC, E-MTAB-1328, CPTAC-3)

**
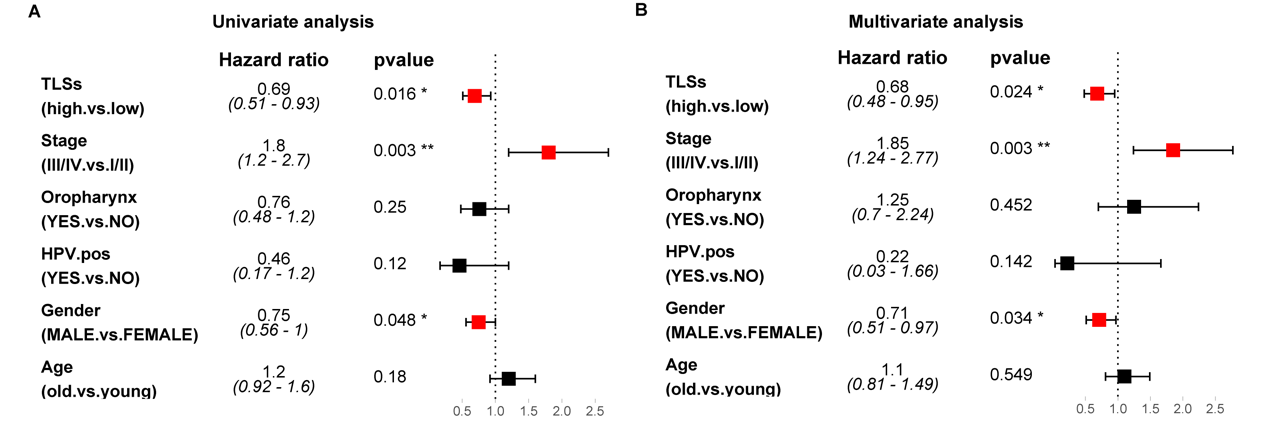
**

**Figure S2**. The univariate and multivariate analysis of TLS status with relevant risk factors. (Dataset: TCGA-HNSCC)

**
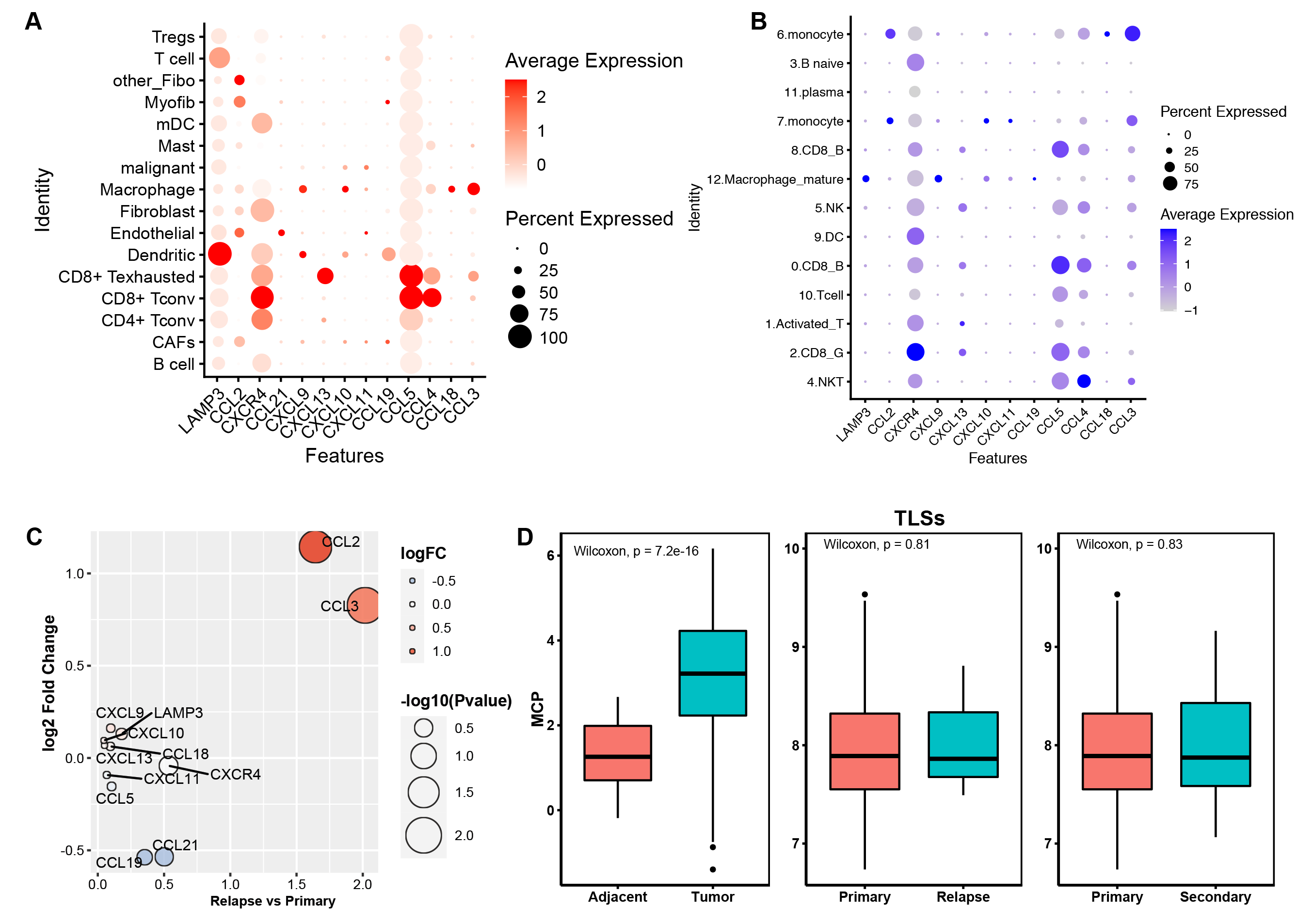
**

**Figure S3.** TLSs distribution between different type of tumors. (A) Expression profiling of selected 13 chemokines in HNSCC scRNA-seq data. (B) Expression profiling of selected 13 chemokines in ICB treated scRNA-seq data. (C) Selected 13 chemokines expression between relapse and primary tumors. (D) Difference TLSs level between adjacent and primary tumor, primary and relapse tumor, primary and secondary tumor. (Dataset: single cell data, 2017; GSE65858)


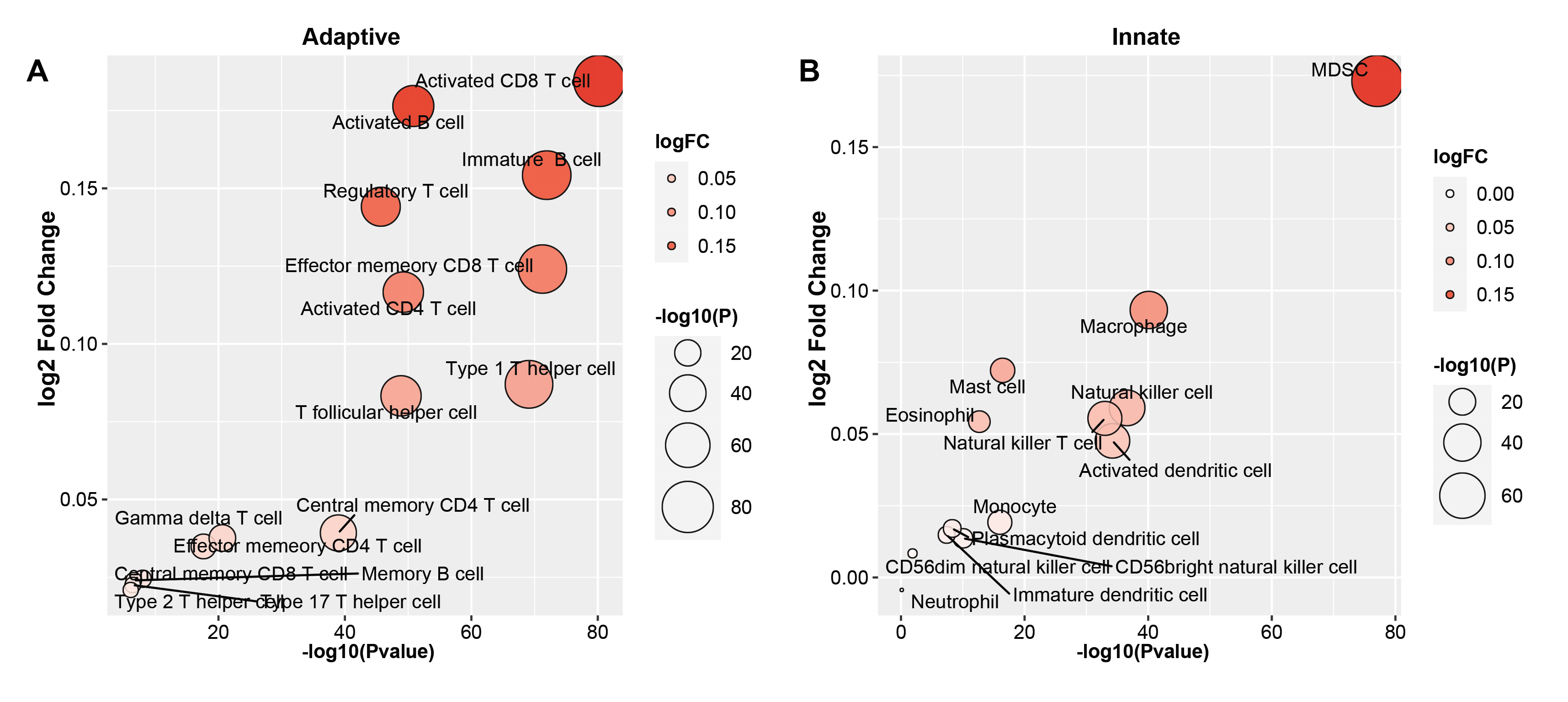


**Figure S4.** Different infiltration of adaptive (A) and innate (B) immunocytes between TLS-hi and TLS-low groups.


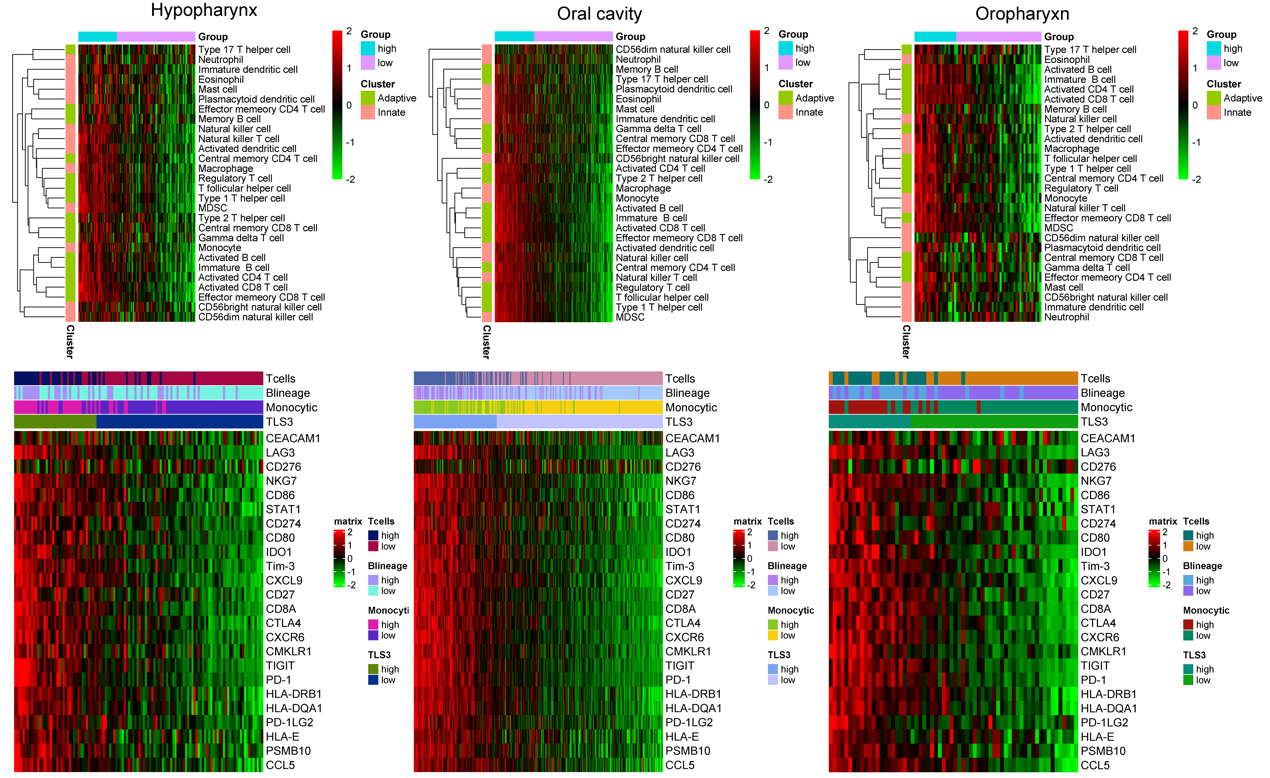


**Figure S5.** The immune cell infiltration and checkpoint expression among hypopharynx, oral cavity and oropharynx cancers.


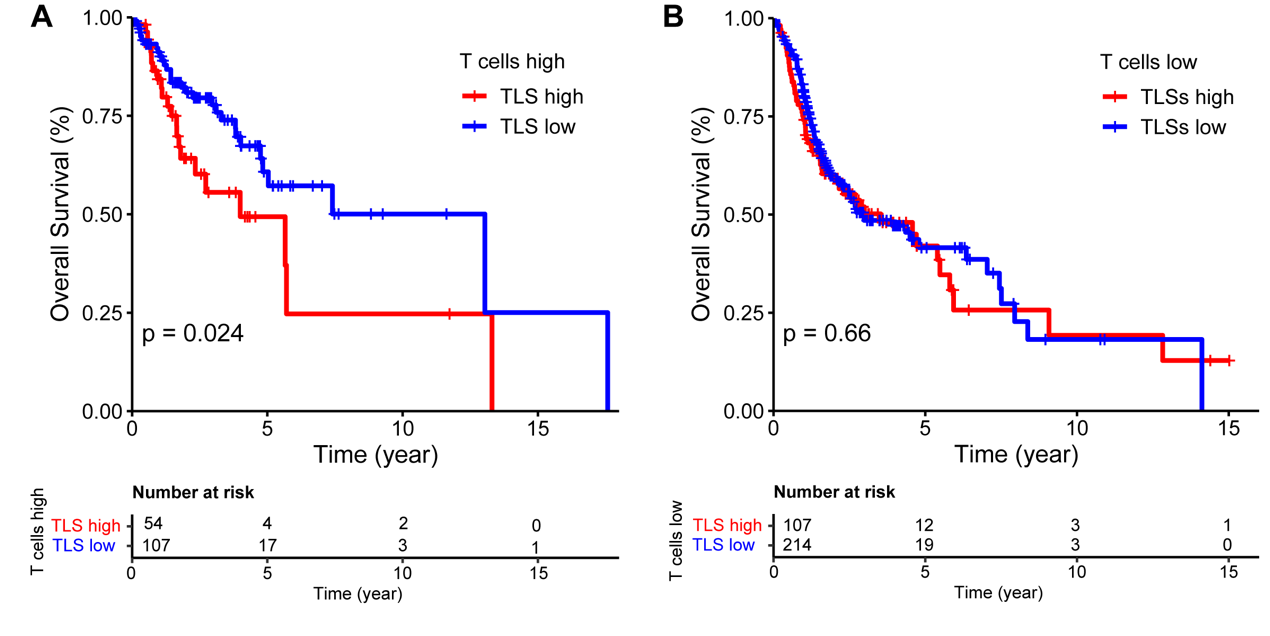


**Figure S6.** The influence of TLS statue on the overall survival of T cell high (A) and T cell low (B) groups. (Dataset: TCGA-HNSCC)


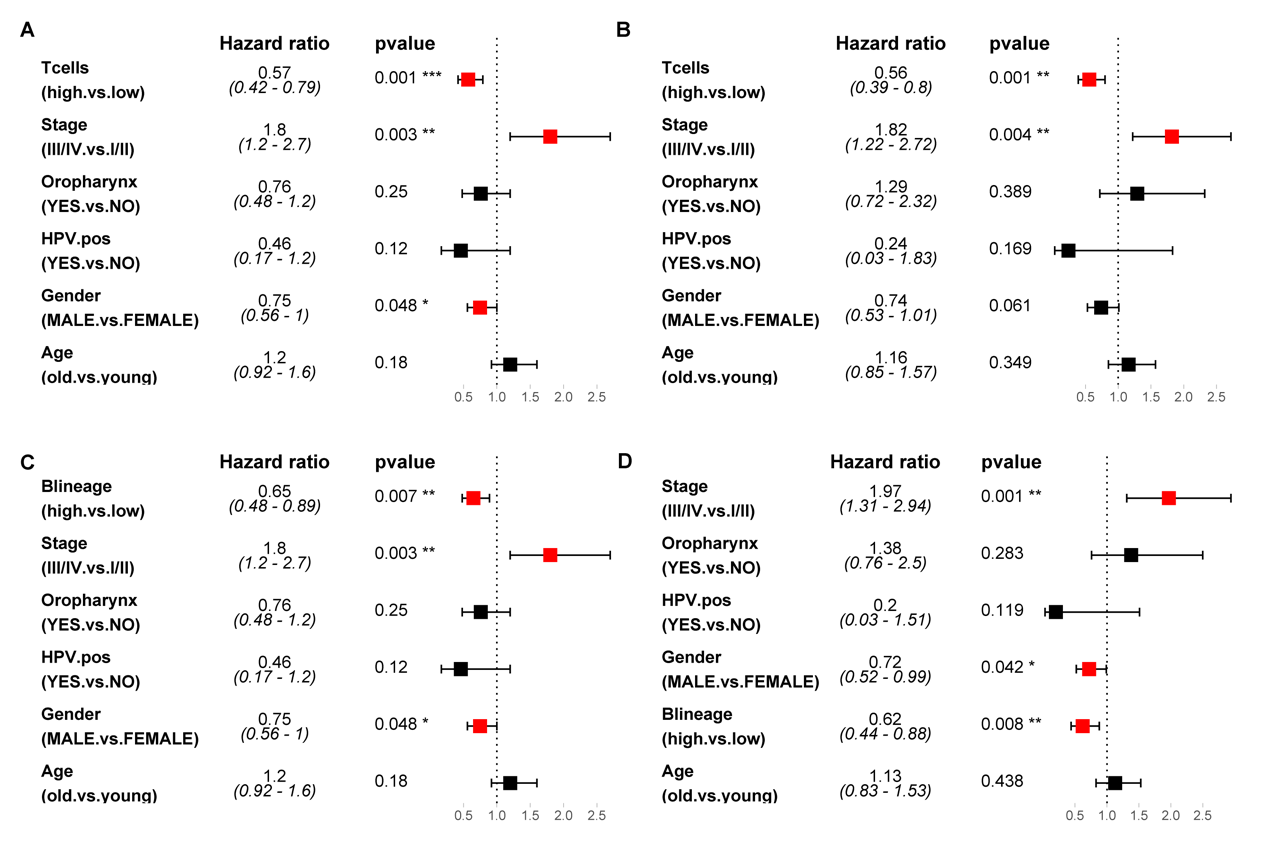


**Figure S7.** The univariate and multivariate analysis of T cell and B lineage with relevant risk factors, respectively. (Dataset: TCGA-HNSCC)


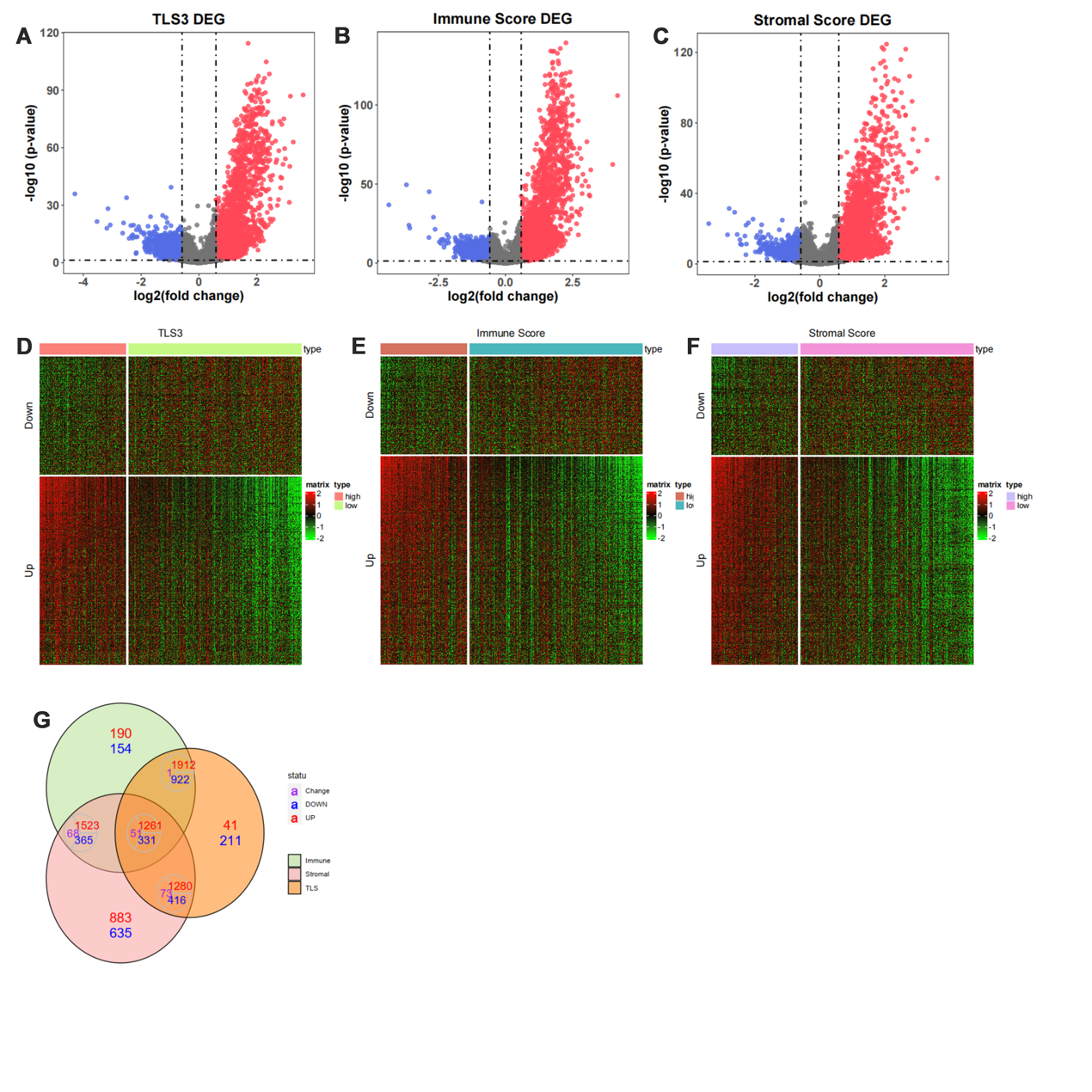


**Figure S8.** Correlation analysis of TLSs with stromal and immune scores. (A) Volcano plot of TLS subtype related DEGs. (B) Volcano plot of immune score related DEGs. (C) Volcano plot of stromal score related DEGs. (D-F) Heat map presentation of TLSs, immune score and stromal score related DEGs. (G) Intersection analysis of DEGs from different subtypes. (Dataset: TCGA-HNSCC)


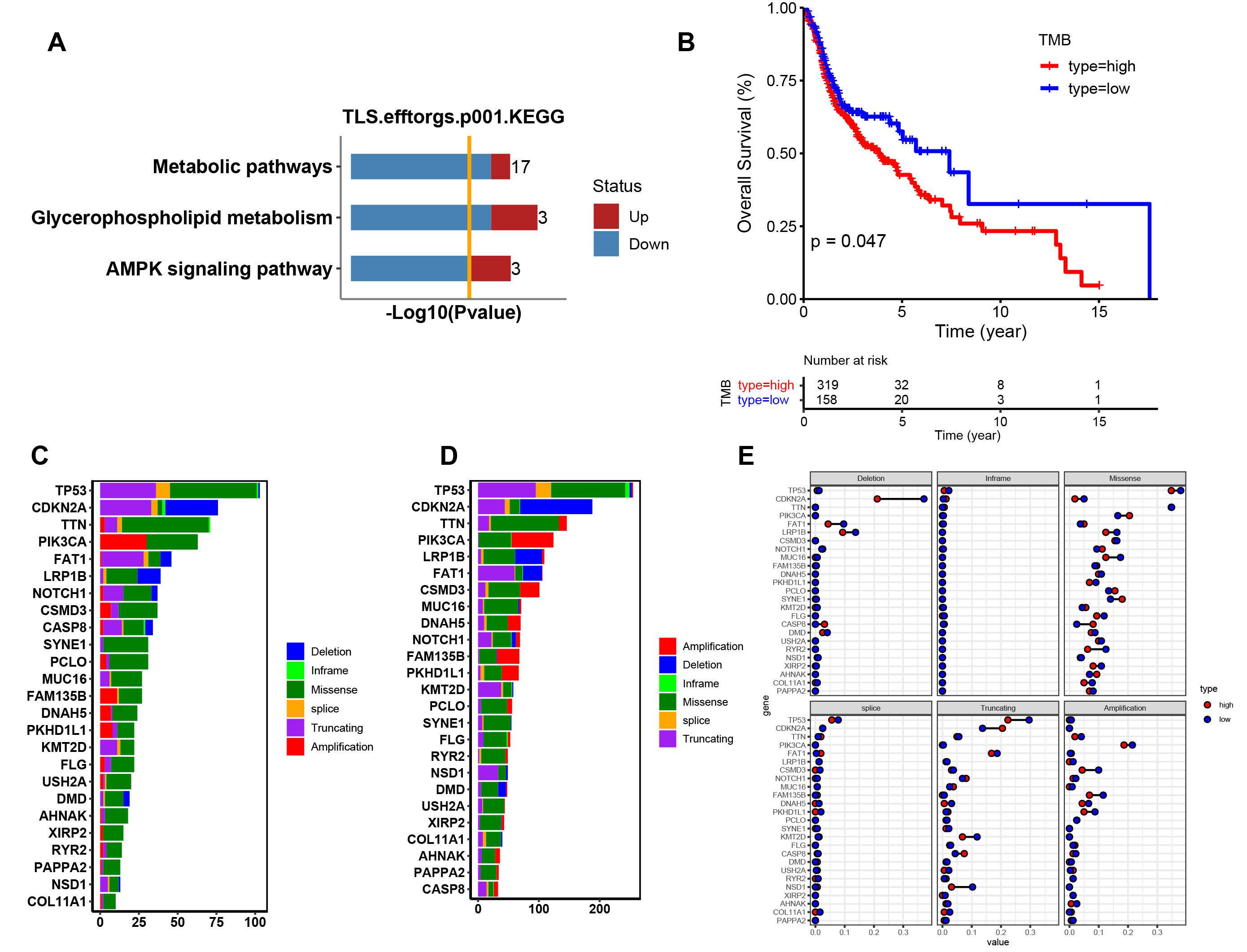


**Figure S9.** (A) KEGG analysis of DEGs between TLS-hi and TLS-low groups under the threshold p value of <0.01. (B) Overall survival analysis of the patients stratified by TMB. (C-D) The highest frequency of mutation of tumor driver gene in TLS-hi and TLS-low groups, respectively. (F) The difference in mutation type of high and low TLSs samples. (Dataset: TCGA-HNSCC)


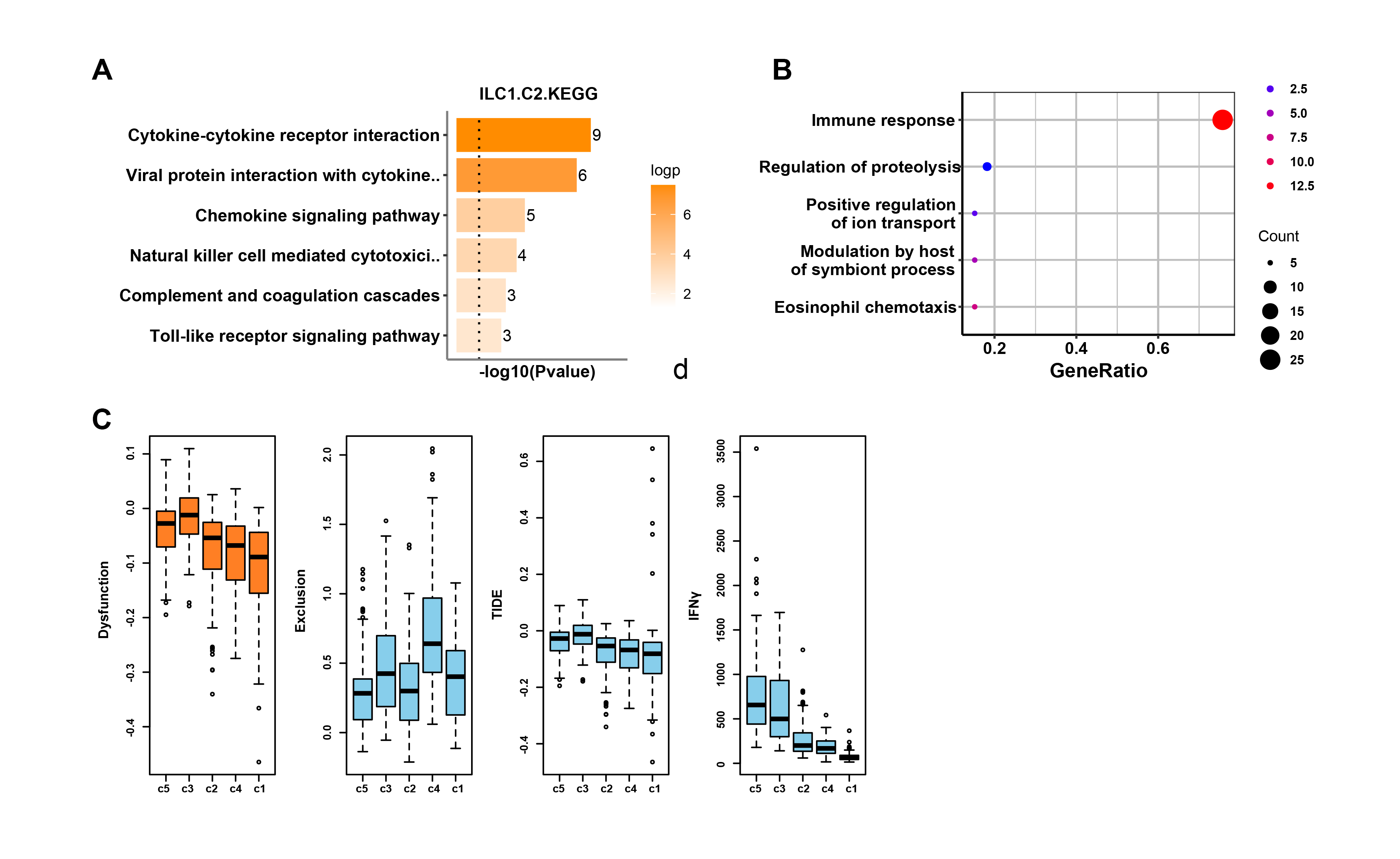


**Figure S10.** (A-B) KEGG (A) and GO (B) enrichment analysis of DEGs of ILC1 between TLS-hi and TLS-low groups. (C) T cell dysfunction, exclusion and IFNγ analysis between TLS-hi and TLS-low groups. (Dataset: GSE137564)

**
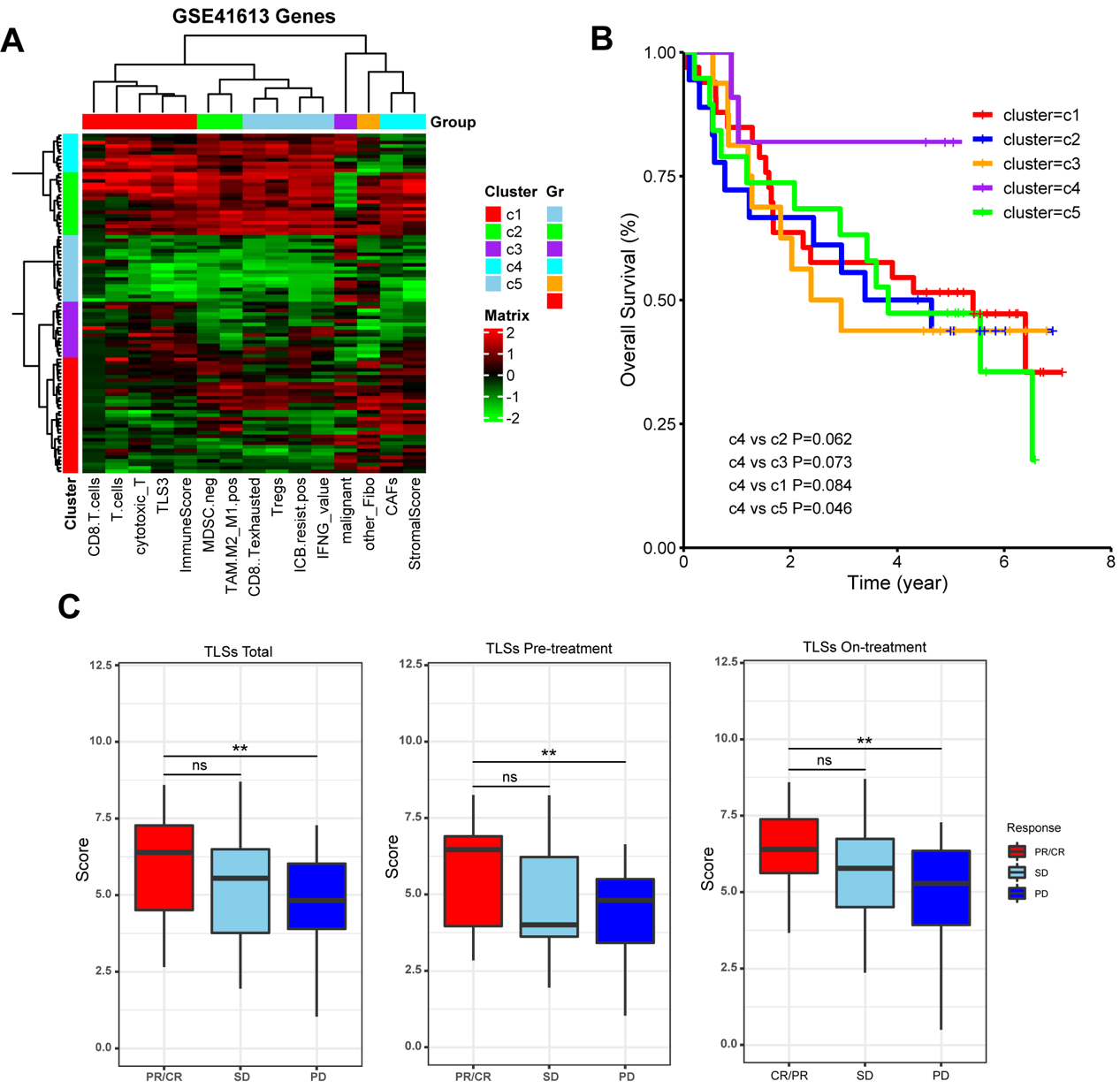
**

**Figure S11.** Reverification of novel TME classification in ICB treatment cohort. (A) Unsupervised clustering of TME parameters. (B) Overall survival analysis of patients within different clusters. (C) TLSs (total, pre-treatment, on-treatment) score distribution in PR/CR, SD, PD patients. (Dataset: GSE41613)

**Figure S12.** The correlation of TLS with tumor progression, EGFR targeted therapy, chemotherapy and radiotherapy.
